# Controlled Drug Release from Nanoengineered Polysaccharides

**DOI:** 10.3390/pharmaceutics15051364

**Published:** 2023-04-28

**Authors:** Ilker S. Bayer

**Affiliations:** Smart Materials, Istituto Italiano di Tecnologia, Via Morego 30, 16163 Genova, Italy; ilker.bayer@iit.it; Tel.: +39-380-387-6699

**Keywords:** drug release, polysaccharide, controlled release, kinetics, nanofibers, nanoparticles

## Abstract

Polysaccharides are naturally occurring complex molecules with exceptional physicochemical properties and bioactivities. They originate from plant, animal, and microbial-based resources and processes and can be chemically modified. The biocompatibility and biodegradability of polysaccharides enable their increased use in nanoscale synthesis and engineering for drug encapsulation and release. This review focuses on sustained drug release studies from nanoscale polysaccharides in the fields of nanotechnology and biomedical sciences. Particular emphasis is placed on drug release kinetics and relevant mathematical models. An effective release model can be used to envision the behavior of specific nanoscale polysaccharide matrices and reduce impending experimental trial and error, saving time and resources. A robust model can also assist in translating from in vitro to in vivo experiments. The main aim of this review is to demonstrate that any study that establishes sustained release from nanoscale polysaccharide matrices should be accompanied by a detailed analysis of drug release kinetics by modeling since sustained release from polysaccharides not only involves diffusion and degradation but also surface erosion, complicated swelling dynamics, crosslinking, and drug-polymer interactions. As such, in the first part, we discuss the classification and role of polysaccharides in various applications and later elaborate on the specific pharmaceutical processes of polysaccharides in ionic gelling, stabilization, cross-linking, grafting, and encapsulation of drugs. We also document several drug release models applied to nanoscale hydrogels, nanofibers, and nanoparticles of polysaccharides and conclude that, at times, more than one model can accurately describe the sustained release profiles, indicating the existence of release mechanisms running in parallel. Finally, we conclude with the future opportunities and advanced applications of nanoengineered polysaccharides and their theranostic aptitudes for future clinical applications.

## 1. Introduction

In biomedical applications, polysaccharides are utilized as demulcents, drug formulations, new-generation dental materials, hemostatics, in dusting powders, and for the treatment of mild intestinal conditions. Additionally, they can replace plasma and function as anticoagulants both in solution and as surface treatments on artificial organs [1]. As such, polysaccharides are indispensable macromolecules that almost occur in all living organisms and have significant biological functions. They are becoming more important because they exhibit a wide range of biological and pharmacological activities, such as anti-tumor, immunomodulatory, antimicrobial, antioxidant, anticoagulant, antidiabetic, antiviral, and hypoglycemia functionalities that are extremely sought simply after in biomedical and pharmaceutical fields [2,3,4,5,6]. Polysaccharides are polymers of sugars that are monosaccharides linked together by glycosidic bonds. All polysaccharides are formed through the same basic process in which monosaccharides are linked together by glycosidic bonds. The number of carbons in a monosaccharide molecule determines its classification. Triose (three carbons), tetrose (four carbons), pentose (five carbons), and hexose (six carbons) are examples of general categories. The most abundant monosaccharide in nature is hexose or D-glucose. Galactose, which is used to make the disaccharide milk sugar lactose, and fructose, a fruit sugar, are two other common and abundant hexose monosaccharides [7]. An oxygen molecule bridges two carbon rings in these glycosidic bonds. The bond is formed when a hydroxyl group is lost from one molecule’s carbon and a hydrogen group is lost from another monosaccharide’s hydroxyl group. The reaction is a dehydration reaction because two molecules of hydrogen and one of oxygen are ejected. The structure and properties of the resulting polysaccharide are determined by the structure of the molecules being combined. A polysaccharide used for energy storage will allow easy access to the constituent monosaccharides, whereas a polysaccharide used for support will typically consist of a long chain of monosaccharides that form fibrous structures.

There are different types of polysaccharides [7]. A heteroglycan is a polysaccharide that is made up of two or more different monosaccharide units. A diheteroglycan is a polysaccharide that contains two different monosaccharide units; a triheteroglycan contains three different monosaccharide units, etc. Polysaccharides exhibit a molecular structure that can be linear or highly branched, composed of the same (homopolysaccharide) or different (heteropolysaccharide) monosaccharide units. Structural differences confer distinct physical and chemical properties [8]. Carbohydrates serve two key functions: energy and configuration. As energy, they can be simple for fast digestion or complex for storage. Simple sugars are monomers known as monosaccharides. These monomers readily pass through cell membranes and are converted directly into energy. The most significant monosaccharide is glucose (C_6_H_12_O_6_), as it is the desired energy source for cells.

**Figure 1:**
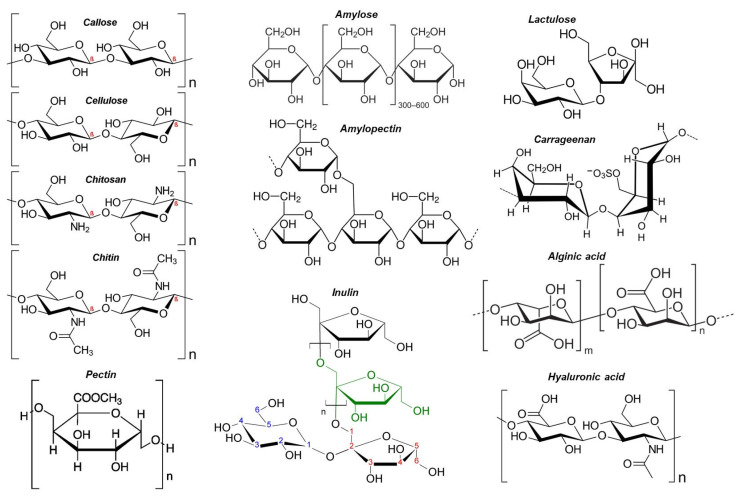
Examples of various polysaccharides and their monomeric units The formulae were reproduced from Wikipedia. The images are ineligible for copyright and therefore in the public domain because they consist entirely of information that is common property and contains no original authorship.

**Figure 2:**
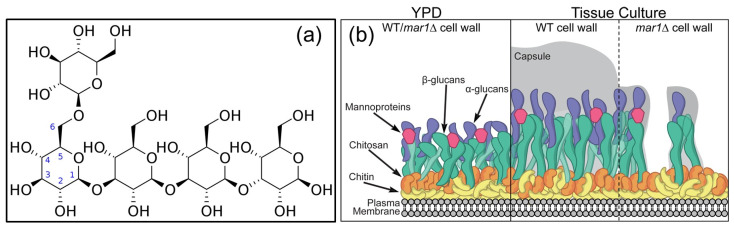
(**a**) A β-1,6 glucan molecule showing how carbons are numbered. The terminal saccharide is linked via a β-1,6 glycosidic bond. The remaining linkages are all β-1,3. The formula was reproduced from Wikipedia. This image is ineligible for copyright and therefore in the public domain because it consists entirely of information that is common property and contains no original authorship. (**b**) A schematic of the structural polysaccharides of the fungal cell wall, which include alpha/beta glucan combined with chitin and chitosan to form the fungal cell wall [5].

Polysaccharides are broadly divided into two types: Homo-polysaccharides are composed of a single type of monosaccharide unit. For example, cellulose, starch, and glycogen (see Figure 1). Hetero-polysaccharides are polysaccharides composed of two or more types of monosaccharide units. Hyaluronic acid, for example, affords extracellular support for organisms. Polysaccharides are typically synthesized using one of three methods: (1) step-wise glycosylation; (2) condensation polymerization; and (3) ring-opening polymerization. However, unlike nucleic acids and proteins, which can be easily synthesized using commercially available automated synthesizers, the simple chemical synthesis of polysaccharides with well-defined structures remains an unsolved challenge. The main reasons are the difficulties in controlling the regioselectivity of multiple hydroxyl groups with similar reactivity, controlling the stereochemistry of glycosidic linkages, and obtaining high molecular weight polysaccharides [8]. A glycosidic bond, also known as a glycosidic linkage, is an ether bond that connects a carbohydrate (sugar) molecule to another group, which may or may not be another carbohydrate. A glycosidic bond is formed when the hemiacetal or hemiketal group of a saccharide (or a molecule derived from a saccharide) and the hydroxyl group of another compound, such as alcohol, come together. A glycoside is a substance that contains a glycosidic bond. The relative stereochemistry of the anomeric position and the stereocenter farthest from C1 in the saccharide can be used to distinguish between α- and β-glycosidic bonds when an anomeric center is involved in a glycosidic bond (as is frequently the case in nature; see Figure 2a). Pharmacologists frequently glucuronidate substances to increase their water solubility by attaching them to glucuronic acid via glycosidic bonds. Numerous other glycosides serve crucial physiological purposes. The fungal cell wall, for instance, is a critical structure with high plasticity that is essential for cellular integrity and viability. The cell wall regulates many biological functions, including controlling cellular permeability and protecting the cell from osmotic and mechanical stress. The cell wall is mainly made up of glucans, chitin, and glycoproteins, as shown in Figure 2b.

Polysaccharides of various types, such as cellulose and its derivatives, chitin and chitosan, hyaluronic acid, alginate, and pectin, have been used in a variety of applications, including tissue engineering, drug delivery systems, facemasks, and bio-sensing [9,10,11,12,13,14,15]. They are manufactured or transformed into a variety of forms, including hydrogels, nanoparticles, membranes, and porous drug delivery media. 

**Figure 3:**
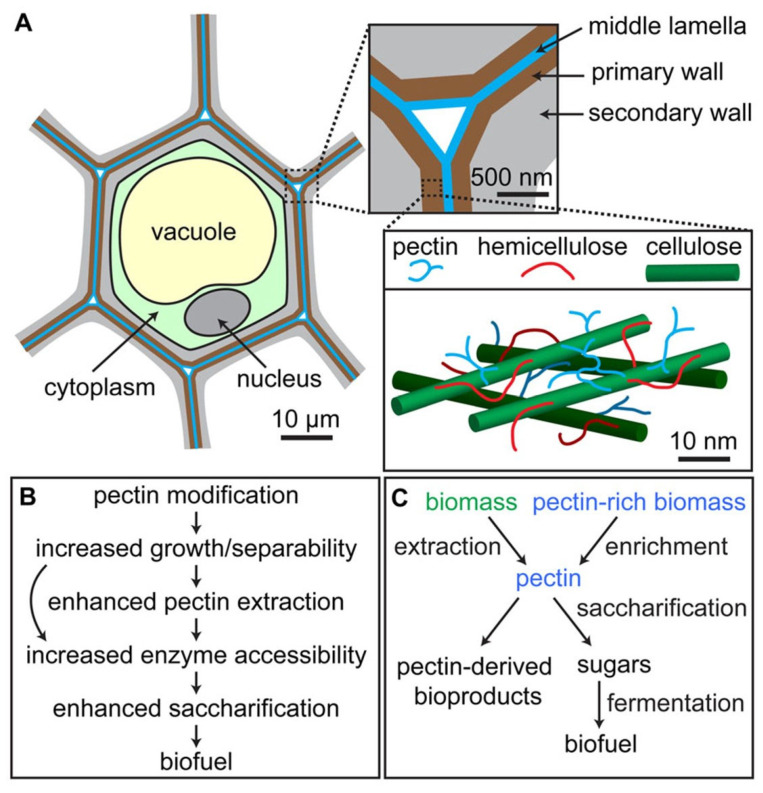
(**A**) Schematic representation of plant cell wall morphology and pectin synthesis. Pectin is abundant in the primary walls that growing cells create (brown) and the middle lamella that binds neighboring cells (blue), but it is also present in smaller amounts in secondary walls that are created after growth has stopped (gray). A simplified representation of the primary cell wall is shown in the inset at the lower right, where cellulose microfibrils (green), hemicellulose (red), and pectin (blue) are arranged in one potential configuration. (**B**) Pectin-rich biomass can be produced from the lignocellulosic feedstock or naturally pectin-rich plant matter, which can then be processed into high-bioproductsoducts derived from pectin or saccharified and fermented to produce biofuel. (**C**) Potentially advantageous effects of pectin modification on biomass processing in bioenergy crop plants. In some circumstances, pectin modification might make it possible to skip steps in the processing process like pectin extraction (curved arrow in (**B**)). Reproduced with permission from [19].

Polysaccharides that contain both hydrophobic and hydrophilic moieties in their molecular chains are referred to as amphiphilic polysaccharides. Furthermore, because polysaccharides frequently contain a large number of -OH (hydroxyl) and/or -COOH (carboxyl) groups in their formulae, extra functional groups can be covalently introduced through chemical modification methods such as sulfation, methylation, carboxymethylation, acetylation, selenylation, and etherification [16,17,18]. It must be acknowledged that even though polysaccharides have many chemical complexities, it is possible to propose a chart that can be used to classify various types of polysaccharides, as shown in Table 1 [16,17,18]. Certain polysaccharides concurrently possess several structural characteristics, such as pectin. Pectin is both plant-derived and negatively charged, and it has branched polymeric architecture [19,20]. Various polysaccharides, including cellulose, pectin, and hemicellulose, are combined to form the composite structures that make up plant cell walls. Pectin is one of them and is continually produced during cellular expansion. It has been hypothesized that pectin contributes to cell adhesion because it is prevalent in intercellular spaces. In food, pectin also serves as a gelling agent. Researchers have attempted to identify and categorize the enzymes involved in pectin synthesis (see Figure 3), as well as establish the mechanisms of pectin synthesis [21], in order to reveal the function of pectin, which is essential to both plants and humans. In fact, many controlled-release formulations based on hydrophilic matrices have chosen pectin due to its non-toxicity and low production costs (Figure 3), making it a common biomaterial for the formulation of controlled-release dosage forms [22].

As medical polysaccharides derived from plant sources, mushrooms encompass an immense and yet fundamentally untapped source of powerful new pharmaceutical products. In modern medicine, they can provide unlimited polysaccharide sources with antitumor and immunostimulating properties [23,24,25]. These polysaccharides are of different chemical compositions and are composed of β-glucans that have β-(1→3) linkages in the main polymeric glucan with additional β-(1→6) branches that are crucial in antitumor action. As such, certain polysaccharides are direct and effective drugs. Table 2 summarizes commonly available polysaccharide-based drugs along with their biological effects and applications [26,27,28].

As shown in Table 2, arabinogalactan, galactomannan, and pectic polysaccharides derived from higher plants, β-glucans, and glycoproteins derived from mushrooms, and sulfated polysaccharides derived from seaweed all have antioxidant and immunomodulatory properties [26]. Heparin is an anionic glycosaminoglycan with a broad molecular weight distribution and charge density that is heterogeneous, linear, and highly sulfated. As a result, heparin can selectively interact with multiple proteins, resulting in a variety of pharmacological functions such as anticoagulant, anti-viral, anti-tumor, and anti-inflammatory properties. Lentinan, which is also included in Table 2, has a β-(1, 3)-glucan backbone with β-(1, 6) branches. It can be extracted from shiitake mushrooms and has been shown to be a biological response modifier for the treatment of gastric cancer. Recent clinical studies show that chemo-immunotherapy with lentinan improves survival in patients with advanced gastric cancer when compared to chemotherapy alone [59]. Finally, high-molecular-weight dextran is a glucose-derived plasma volume expander. It is easily capable of restoring blood plasma loss caused by severe bleeding. Severe blood loss can cause organ failure and brain damage by lowering oxygen levels. Plasma is required for the circulation of red blood cells, which carry oxygen. Dextran is used to treat hypovolemia (low circulating blood plasma volume) caused by surgery, trauma or injury, severe burns, or other causes of bleeding [60].

Certain polysaccharides can be activated by physical or chemical stimuli, and after being triggered, they can facilitate the on-demand burst or controlled release of certain drugs or molecules [61]. For instance, gellan gum, carrageenan, and alginates have been used to construct such response-driven hydrogel-based devices. In addition to various physical or chemical stimuli such as temperature and pH, polysaccharides experiencing chemical stimuli undergo significant changes in their rheological or physical properties. Chemical stimuli can be used to induce changes like self-assembly, sol-gel transition, and hydrogel cross-linking. Most of these changes can lead to the release of molecules or cells, accompanied by matrix swelling or degradation. Aside from chemically induced modifications, certain physical stimuli can also cause in situ modifications of the polysaccharides. A summary of biomedical applications of physico-chemically activated polysaccharides is given in Table 3. As shown in Table 3, swelling and erosion/degradation-induced drug release behavior of certain polysaccharides such as pectin and inulin are of immense importance in controlled release technologies that target sustained release in simulated gastric fluid (SGF) and simulated intestinal fluid (SIF) environments. 

Pectin, for instance, undergoes a pH-dependent hydrogel formation where swelling in SIF can be much greater compared to SGF, releasing only 65% of a model drug over a period of 12 h [77]. This behavior renders pectin-based tablets or biomaterials highly suitable for extended release systems [77,78]. As another example, the sol–gel transition of the mixture of glycol chitosan and oxidized alginate at room temperature can be utilized for the sustained release of Avastin^®^ for ocular drug delivery [79]. Such a material system encapsulating Avastin^®^ can be tuned to burst release at an early stage (within 4 h), followed by a sustained release extending to several days. The sol-gel transformation can be tuned by increasing the oxidized alginate concentration in the hydrogel [79]. Note that oxidized alginate gels were shown to be cytocompatible for a variety of cell types, including corneal endothelial cells. The swelling behavior, degradation profile, and storage modulus of these modified alginate gels are all affected by alginate oxidation, allowing these properties to be controlled depending on the degree of oxidation. Moreover, oxidized alginates are better soluble under physiological conditions, allowing better gelation through dynamic covalent Schifft-base linkage between amino groups on chitosan [79]. 

## 2. Principles and Kinetics of Drug Release: A Brief Review

Drug release from encapsulating matrices is a very complicated process that can depend on matrix material chemistry, structure (such as porosity, gel state, etc.), medium conditions such as pH, presence of enzymes, etc., along with external triggers such as temperature. Drug release takes place in several different modes, such as systems that release a drug at a slow, i.e., zero or first-order rate, and those that provide an initial rapid dose, followed by a slow zero or first-order release of sustained components [80]. Certain sophisticated systems have also been developed suitable for pulsatile drug release [81,82]. The purpose of controlled release systems is to conserve desired drug levels in the blood or in target tissues for as long as possible. In other words, controlled release matrices must regulate the drug release rate and duration in an anticipated medium. As such, an initial faster release or dose is needed in order to rapidly accomplish the effective therapeutic concentration of the drug. Then, well-defined drug release kinetics must be followed in order to supply and maintain medical doses and drug levels over a desired period [83]. Table 4 shows the most commonly used mathematical models for evaluating drug release kinetics that are generally fitted to the experimental release data.

**Figure 4:**
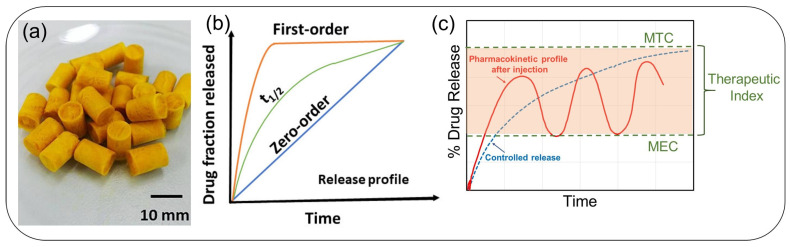
(**a**) Macroscopic appearance of alginate-based sponges loaded with curcumin-loaded self-micro-emulsifying drug delivery systems [83]. (**b**) Exemplary drug release profiles and (**c**) illustrative representation of the release pattern of the drug in immediate-release dosage (red curve) and controlled release (dashed blue). MTC: Maximum therapeutic (tolerated) concentration. MEC: Minimum effective concentration.

A plot of plasma drug concentration versus time is a common way to represent the drug release profile. The minimum effective concentration, below which the drug is ineffective, and the toxic concentration, above which undesirable side effects occur, are both depicted in the plot of Figure 4. Preservation of drug concentration at any instance between the minimum effective concentration and the minimum toxic concentration is crucial for safety and therapeutic effectiveness [83]. Drug release kinetics is zero-order when a constant amount of drug is excluded per unit time, but the rate remains independent of the concentration of the drug. Several medications are administered in immediate-release forms, which result in a quick rise in systemic drug concentration. These formulations may have limitations due to poor patient compliance, harsh side effects, low bioavailability, or unfavorable pharmacokinetics, despite the fact that they have historically played a significant role. Although first-order release kinetic drug delivery has been able to improve pharmacokinetics, it is still not the best option for medications with brief biological half-lives or narrow therapeutic windows. Zero-order release has the potential to overcome the issues faced by immediate-release and first-order systems by emancipating the drug at a constant rate, hence maintaining drug concentrations within the therapeutic zone for an extended period [83,84,85,86].

Figure 4a exemplifies alginate-based porous sponges to be digested as carriers to prolong the gastric retention time and controlled release of curcumin. Curcumin was incorporated into the sponges via an emulsion process [83], and the final composite was converted into a solid form by freeze-drying [83]. The curcumin release from these sponges followed the Higuchi profile ($Q_{t}=K_{H}t^{0.5}$) as shown in Figure 4b as a *t*_1/2_ curve, which translates into a sustained release that was maintained for over 8 h. Figure 4c shows a plot of plasma drug concentration versus time. There are two important concentration levels indicated: the minimum effective concentration (MEC), below which the drug is ineffective, and the maximum therapeutic (tolerated) concentration, above which unwanted side effects can occur. Maintaining drug concentrations between the minimum effective concentration and the minimum toxic concentration is critical for safety and therapeutic effectiveness. Table 4 summarizes a number of popular drug release models developed over the years to design and study new drug release matrices. The model examples in Table 4 can be briefly described as follows:

- Zero-order: By releasing medication at a constant rate and keeping drug concentrations within the therapeutic window for a longer period, zero-order drug delivery systems can solve problems with immediate-release and first-order systems. This release profile can be used to lower dosage requirements, lessen dosing intervals, and improve receptor binding, post-receptor effects, and chemical interactions in terms of pharmacodynamics [87];
- First-order: Various therapeutic agents’ absorption and/or elimination have been described using this model. However, using a basic theory to define first-order kinetics is challenging. In this sense, first-order release states that the kinetic release rate depends on how the drug concentration changes over time [88];
- Higuchi model: The model defines drug release from insoluble matrices as a function of the square root of time, related to the Fickian diffusion equation. The slope of the plot gives the Higuchi dissolution constant [89];
- Hixson-Crowell model: This is a cube root law that deals with the dissolution rate that is normalized with respect to the decrease in solid surface area as a function of time. Adaptable to matrices where there is a change in the surface area and diameter of particles or tablets. It assumes no shape change as the suspended solid dissolves; its surface decreases by two-thirds of its weight [90];
- Baker-Lonsdale: It is a modified Higuchi model and describes the drug release from spherical matrices [91];
- Korsmeyer-Peppas model: This model was established specifically for the release of drugs from polymeric matrices like hydrogels [92]. As a power law, a comprehensive semi-empirical equation that establishes an exponential relationship between the release and the time. Modified forms have also been employed that contain the latency time, which marks the launch of drug release from the matrix;
- Hopfenberg model: It models and correlates drug release from surface-eroding polymers and assumes that the surface area remains constant during the degradation process. Good for drug release from slabs, spheres, and infinite cylinders displaying heterogeneous erosion [93];
- Poiseuille’s law of laminar flow. It can model drug release from membrane matrices, such as monolithic osmotic tablet systems. It is used for drug release from swelling gels or tables through orifices via pressure difference [93];
- Fickian drug release from Euclidian or fractal matrices can be modeled or approximated by the Weibull function. It is also used to model dissolution-induced drug release [94,95].

Drug transport from matrices includes multiple steps driven by different physical or chemical phenomena, making it challenging, or even impossible, to implement a mathematical model describing it in the right way. The most widely reported drug release models are the Higuchi model, the zero-order model, the first-order model, and the Korsemeyer-Peppas model. Note that the physicochemical properties of the drug as well as the polymer and the drug-to-polymer ratio dictate drug release dynamics from the formulation, and in many cases, more than one release mechanism can occur during the administration period, particularly from nanoscale carriers [95].

## 3. Drugs and Their Properties Encapsulated by Polysaccharides

Drug release studies based on polysaccharides utilized a vast variety of model drugs, ranging from antiseptics and antibiotics to antioxidants, painkillers, and proteins [95,96,97,98,99,100]. Table 5 demonstrates some examples of drug-encapsulated pure or modified polysaccharides [101,102,103,104,105,106,107,108,109,110]. For instance, chitosan—tamarind seed polysaccharide composite films were evaluated for the delivery of protein/peptide molecules through transdermal transport. The blend constituent concentrations were adjusted to tune the solubility, pH resistance, and swelling properties of protein encapsulating matrices [103]. In another study in Table 5, a pH-sensitive biodegradable ternary blended hydrogel film (chitosan/guar gum/PVP) was developed for antibiotic delivery applications [109]. Different degrees of crosslinking with sodium tripolyphosphate resulted in controlled swelling and release of the antibiotic under different pH conditions, like gastric and intestinal fluids. Probably the most frequently used model drugs are antibiotics and cancer drugs. Hence, it is important that the readers are familiarized with their properties, functions, and targeted clinic applications. 

Antibiotics are generally chosen as model drugs in many polymer-matrix release formulations. Extended-release dosage forms maximize the therapeutic effect of antibiotics while reducing antibiotic resistance by maintaining a constant plasma drug concentration over MEC for an extended period (Figure 4c). Table 6 lists some common antibiotics utilized as model drugs in polysaccharide release studies, along with their target actions and potential side effects [111,112].

Moreover, Table 7 lists commonly used antibiotics to treat a variety of cancers, ranging from soft tissue tumors to Ewing’s sarcoma (a rare bone tissue cancer) [113]. Almost all antibiotics displayed in Table 6 and Table 7 have been incorporated into polysaccharide-based controlled-release formulations. Encapsulation of antibiotics in nano-engineered polymer matrices is considered effective against the surge of antibiotic-resistant bacteria.

By crossing the cell membrane, interfering with cellular elements, and harming bacterial metabolism, antibiotics in nanoscale matrices can act as carriers and delivery agents to reach target sites inside bacteria. Antibiotics carried in polysaccharide-based nanocarriers often display high encapsulation efficiency. The distinct characteristics of such drug carriers in terms of size, shape, and composition present bacteria with multiple simultaneous threats, and it is expected that bacterial resistance to various nanoscale conjugates develops much more slowly [113,114,115]. In addition to physically destroying bacteria, nano-polysaccharides with antibiotics also disrupt key molecules involved in bacterial processes, and hence genetic mutations from abiotic attacks wielded by such systems are less probable. Hence, it is important to briefly exemplify some selected works that incorporated model antibiotics in nanoscale polysaccharides. Among various antibiotics, for instance, vancomycin-incorporated chitosan nanoparticles have been developed for contact lens applications [114]; vancomycin was also incorporated in natural polysaccharide-based hybrid hydrogels for synergistic wound fumigation using low-intensity near-infrared light-triggered spatiotemporal antibiotic release and hyperthermia [115].

**Figure 5:**
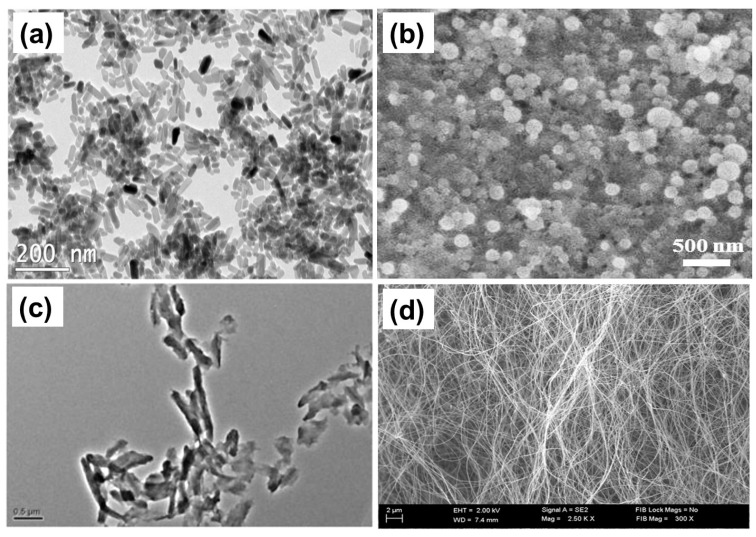
(**a**) Alginate modified apatite nanoparticles [118], (**b**) caseinate-zein-polysaccharide complex nanoparticles for hydrophobic drug encapsulation [119], (**c**) methotrexate-conjugated pectin nanoparticle bundles [120] and (**d**) typical nanofibrillar structure of bacterial cellulose [124].

Other recent works, for example, studied chloramphenicol release dynamics of poly (vinyl alcohol)/sodium alginate hydrogels made by freeze-thaw and calcium ion crosslinking to imitate gastrointestinal tract conditions and assess the clinical practicability of the hydrogels as controlled-release drug carriers [116]. Hydrogels of pectin prepared by the reaction between pectin and bis (3-aminopropyl) amine (APA) exhibited a pH-induced sol-gel phase transition and were found to regulate the release of gentamicin [117]. Controlled antibiotic release for the management of periodontal infrabony defects using bioactive gelatin-alginate/apatite nanocomposite films has been demonstrated using tetracycline [118]. A typical nanoscale structure of apatite nanoparticles is shown in Figure 5a. Caseinate-zein-polysaccharide complex nanoparticles (Figure 5b) can easily encapsulate hydrophobic drugs, and via polysaccharide type and chemical cross-linking, the drug release rate can be regulated [119]. As mentioned briefly before, pectin, as a natural polysaccharide, exhibits interesting properties for drug delivery, particularly in the form of nanoparticles [120] (Figure 5c).

A notable study, for example, synthesized methotrexate-conjugated pectin nanoparticles for delivering a cytotoxic drug to hepatic cancer cells. Carbodiimide chemistry was used to conjugate methotrexate with pectin. The nanoparticles demonstrated sustained drug release at pH 7.4, while methotrexate’s cytotoxicity was increased when conjugated to pectin nanoparticles versus free methotrexate [121]. Table 7 exemplifies a number of model cancer drugs that have been incorporated into polysaccharide release matrices. Alginate nanoparticles carrying paclitaxel (PTX) were modified with chitosan and folate-chitosan using a double emulsion cross-linking electrostatic attraction method. The nanoparticles were between 200 and 300 nm in size, and they were tested for in vitro anti-cancer activity and cellular uptake by HepG2 cells. The results revealed that the modified nanoparticles allow sustained release of anticancer drugs while having no cell toxicity [122].

It is also important to mention that bacterial nanocellulose is an effective antibiotic delivery system that is nontoxic and highly biocompatible. It has been extensively utilized as a nanostructured (i.e., nanofiber networks shown in Figure 5d) cancer drug carrier and delivery vehicle in addition to its use as a matrix for improved therapeutic potency while reducing the adverse effects of chemodrugs by decreasing their dosages [123,124]. Another common antibiotic incorporation method into polysaccharides is the solid dispersion technique, which appears to increase the dissolution rate and bioavailability of poorly soluble drugs. The solid dispersions have unconventional uses in the area of controlled release dosage matrix design because of the wide availability of certain polysaccharides that are partially soluble or have excessive swelling capacity in aqueous media, such as ethyl cellulose, hydroxypropyl cellulose, and hydroxypropylmethyl cellulose [125]. Additionally, exceedingly precise physio-chemical surface modification techniques to create the ideal polysaccharide nano-carriers should be made with more biocompatible procedures to tailor to the drug properties but also eliminate associated toxicological concerns related to surface functionalization moieties.

## 4. Polysaccharide Encapsulated Natural Extracts and Release

It is projected that over 60% of the accepted drugs and new drug developments for cancer and infectious diseases will be based on natural extracts [126]. Complex plant-derived combinations, such as natural extracts, are highly challenging to characterize and study to document their pharmacological potency properly. However, many natural extracts feature a multi-targeted mode of action and impending synergistic performance on different proteins within the same signaling pathway and in several biochemical pathways at once, rendering them very attractive for anticancer drug development [126]. Natural extracts, including essential oils, have been incorporated into polysaccharide matrices for biomedical applications, including sustained drug release [127,128,129,130,131,132,133]. Curcumin, for instance, has been extensively used in polysaccharide nanocarriers as it is a very difficult agent to metabolize due to its lipophilic nature [134,135]. Another interesting example is the system in the form of a polymeric film based on chitosan containing aqueous mint and pomegranate peel extracts that have potent antibacterial activity against Staphylococcus aureus and Bacillus cereus [136]. Controlling the release of phenolic compounds as natural antioxidants has a major role in their antioxidant activity. To this effect, recent studies have utilized nanoscale encapsulation techniques for, for instance, olive leaf phenolic compounds through pectin complexes to evaluate their release rate [137]. Efficient nanoscale encapsulation of these extracts enabled their controlled release for up to 20 days in various biological fluids. The study by Ahmed et al. [138] screened about 23 wild plant extracts along with 24 spice and herb extracts using ethanol and water. Ethanoic and water-suspended natural extracts were tested in vitro as anticancer agents employing the trypan blue technique against Ehrlich Ascites Carcinoma Cells (EACC), while the sulphorhodamine (SRB) assay technique was used against HepG2 cells. The antioxidant potency of the extracts was measured by 2, 2 diphenyl-1-picrylhydrazyl (DPPH) assay. Based on the summary of their results shown in Table 8, both ethanoic and water extracts of some plants featured high cytotoxic and antioxidant action and inhibited the growth of cancer cells. 

More specifically, the anticancer actions of 35 plant extracts indicated that 17 ethanoic and 18 aqueous extracts provided anticancer activity greater than 70% (Table 8). The highest inhibition (100%) was detected in the ethanoic extracts obtained from *Solanum nigrum*, *Atriplex* sp., and *Astragalus spinosus*, followed by *Arum palaestinum* (97.29%), as shown in Table 8.

Essential oils are of utmost significance in the pharmaceutical, cosmetic, agricultural, and food industries [139]. However, essential oils’ potency is based on their bioactivity and stability. An established method for the preservation of essential oils is polymeric nanoscale encapsulation. Numerous advantages are available, such as increased water solubility, efficient defense against deterioration, avoidance of volatile component evaporation, and controlled and directed release. Nanoprecipitation is one of the many methods used to create polymeric nanoparticles, and it has garnered a lot of interest. The most notable contribution of nanotechnology to encapsulating essential oils is known as the nanoprecipitation method [140]. Chitosan is a common encapsulation polymer due to its abundant natural supply, biodegradability, and free NH_2_ surface functional groups. The NH_2_ groups on the chitosan surface make it simple to graft functionalized molecules such as quaternization, which is frequently accomplished in an acidic environment. Quaternization strengthens the ionic character of chitosan. Despite the fact that chitosan’s cationic nature alone exhibits antibacterial activity due to its interaction with negatively charged bacterial membranes, the material’s nanoscale size further enhances its antibiofilm activity [141]. Electrospinning is also a very common method to encapsulate essential oils in chitosan, while there are several solubility issues associated with other polysaccharide polymers [142].

Phenolic compounds are imperative micronutrients in our diet, and there is growing evidence that they may prevent degenerative illnesses like cancer, inflammation, and neurodegenerative diseases [143,144]. The main constraints on the use of phenolic compounds are their low bioavailability and ease of destruction under environmental stresses. It has been suggested that polysaccharide nano-encapsulated phenolics can be protected better and metabolized easier with high efficiency [143]. For example, polyphenols may interact with starch and be transported through the digestive system as a result. The starch-based carriers can deliver polyphenols with targeted or sustained release after physicochemical or enzymatic modification [144,145]. Table 9 demonstrates a number of selected examples of phenolic compounds like curcumin encapsulated in nanoscale or sub-micron-scale polysaccharide structures that are tailored towards improving their bioavailability and sustained release through the skin or through gastrointestinal mechanisms.

More encapsulating edible polymer systems have been reviewed in [156], however, with a major focus on improving the physicochemical and functional properties of food or food packaging. Additionally, a comprehensive review on the application of nano/microencapsulated phenolic compounds against cancer [157] indicated the importance of targeted sustained release of phenolic compounds but did not present and discuss sustained release dynamics and pharmacokinetics of these compounds in nanoscale polysaccharide matrices.

For instance, very few studies exist that demonstrate the detailed release analysis of phenolic compounds from nanoscale pectin matrices. It has been shown that pectin-decorated selenium nanocarriers of curcumin can accomplish enhanced physicochemical and biological properties [158], and the authors used the Ritger-Peppas model to conclude that an anomalous (non-Fickian) drug transport mechanism was achieved from their nanoparticles. In another recent work, functionalized cellulose-based nanocarriers were fabricated by applying an acid-alkali treatment to cellulose. Folic acid was then conjugated with the nanocellulose. For the controlled delivery of curcumin, glycidyl methacrylate (GMA) and hydroxyethyl methacrylate (HEMA) were polymerized with folic acid and conjugated to the nanocellulose matrices. The authors used the Korsmeyer-Peppas kinetic model and concluded that their release mechanism followed drug diffusion and simultaneous polymer matrix swelling [159]. Using various mathematical models like first-order, zero-order, Hixson Crowell, Korsmeyer-Peppas, and Higuchi, Kumari et al. [160] studied curcumin release from lemongrass cellulose nanofibers at various simulated pH conditions. In all of the simulated pH conditions, it was found that curcumin release was best fitted to the Korsmeyer-Peppas equation with an *R*^2^ value of 0.94 (pH 1.2), 0.96 (pH 5.3), 0.92 (pH 6.8), and 0.94 (pH 7.4). The diffusional exponent, or *n* value, is related to the drug transport mechanism. If n < 0.45, Fick’s diffusion mechanism prevails, and for *n* values between 0.45 and 1, the non-Fickian diffusion mechanism takes over. For all the pH values studied, the *n* value remained below 0.25, and the authors concluded that since curcumin was adsorbed on the nanocellulose surface via Langmuir and Freundlich isotherms, indicating coexistence of homogeneous monolayers and heterogeneous multilayers over the nanofibers, curcumin was released via swelling and diffusion [160].

Other natural extracts based on animal feedstock, such as ovalbumin, have also been used for controlled release studies encapsulated in chitosan or carrageenan nanoparticles, and release for up to 21 days could be maintained [161]. In another study [162], bovine serum albumin was used as a model drug to systematically examine the applicability of bacterial nanocellulose as a drug delivery matrix. Freeze-dried and never-dried matrix forms were made and tested for drug release. For albumin release from both nanocellulose matrix systems, a dependence on concentration, temperature, time, and pre-swelling was shown. The results suggested an overlap of diffusion- and swelling-controlled release that could be described by the Ritger-Peppas equation [162].

## 5. Drug Release from Polysaccharide-Based Nanofibers

One of the most common nanofiber drug release matrices is based on chitosan and its blends of composites with other biopolymers. It is generally claimed that chitosan nanofibers obtained by electrospinning have distinctive properties such as high surface area, good porosity, being nontoxic and biocompatible against cell cultures, being biodegradable and renewable, having low immunogenicity, being inherently antibacterial, and being easy to produce on a large scale [163]. Shikhi-Abadi et al. [163] presented an extensive review on the use of chitosan nanofibers as carriers to deliver anticancer drugs under in vitro conditions. In this section, we will focus on the drug release dynamics, including sustained delivery studies and release model-data comparisons from polysaccharide-based nanofibers encapsulating various drugs and natural extracts. Some studies on polysaccharide-based nanofibers focused on fast or burst release, such as oral films for drug delivery prepared from chitosan or pullulan electrospinning nanofibers that contain aspirin [164]. For instance, tetracycline hydrochloride (TCH) antibiotic-loaded poly(ω-pentadecalactone-co-ε-caprolactone)/gelatin/chitosan nanofibrous membranes were fabricated as a controlled drug delivery system in a very recent work [165]. In vitro drug release studies were then carried out, and mathematical modeling was used to identify the drug transport mechanism. TCH was released over the course of 14 days, starting with a burst in the first hour and then continuing over time. With 96.5% total drug release and 11.8% initial burst release, the 1% TCH-loaded sample was demonstrated to be effective with regard to gram-positive (Staphylococcus aureus and Bacillus subtilis) bacteria, but it had little effect on gram-negative (Escherichia coli) bacteria (no inhibition zone was seen below 3% TCH concentration). The authors implemented four mathematical equations (zero order, first order, Higuchi, and Korsmeyer-Peppas) for the release of TCH from the nanofibrous membranes. The best-fitted mathematical model was selected based on the highest coefficient of determination (*R*^2^). The authors indicated that depending on the release exponent, *n*, the release mechanisms were identified as: *n* < 0.5 pseudo-Fickian diffusion, *n* = 0.5 Fickian diffusion, 0.5 < *n* < 1 non-Fickian diffusion, *n* = 1 case II transport (zero order release), and *n* > 1 super case II transport [165]. It is important to note that the authors therein excluded the initial burst-release data points and used linear fits to the rest of the data. They treated the initial burst dynamics separately, in which about 10% of the drug was released within one hour. Table 10 shows intercepts, slopes, and coefficients of determination (*R*^2^) obtained from linearized fits. High coefficients of determination (*R*^2^ > 0.96) were extracted by applying Higuchi and Korsmeyer-Peppas models, indicating TCH release from the nanofibrous networks was diffusion-controlled. The best fit to the in vitro drug release profiles was Korsmeyer-Peppas (*R*^2^ ∼ 0.98–0.99). The TCH release mechanism was revealed to be pseudo-Fickian-type diffusion because the Korsmeyer-Peppas release exponent (*n*) values were less than 0.5 (between 0.236 and 0.268) for all preparations. The logarithm of the release rate constant was shown as Korsmeyer-Peppas intercept values and is shown in Table 10. The preparations loaded with 0.5% and 1% TCH released drugs the fastest. The authors did not use other potential models such as Weber-Morris (intra-fiber, intra-particle) diffusion that might also describe the release profile in [165].

Understanding the methods to control drug release and how they affect the efficacy of the formulation is just as crucial as understanding the physicochemical mechanisms involved. For instance, drug concentration can be changed to alter the release profile because when it was increased from 0.5% to 5% in [165], the overall drug release decreased from 97% to 42%. Secondly, despite the fact that 1% TCH-loaded nanofibers displayed 97% of the total drug released, no inhibition zone was visible below 3%. The total amount of drug released from the 3% drug-loaded matrix, despite having a drug payload that is three times higher [165], was only 61%. This suggests that the formulation is more effective than the amount of drug it releases. A pseudo-Fickian diffusion mechanism suggested in [165] is similar to the Fickian curve, but the approach to final equilibrium is slower in pseudo-Fickian diffusion. This drug release behavior should not directly influence the formulation’s effectiveness or the total drug released, however slower it will be. In another study [166], a nanofiber drug delivery structure based on polycaprolactone/chitosan blends containing 5-fluorouracil (5FU) was developed. Nanofiber features and drug release performance of various nanofibers were investigated. Increasing the chitosan content in the nanofibers sustained the drug release period. The drug release mechanism from all the nanofibers could be represented by Fickian diffusion based on the Korsmeyer-Peppas model, which is suitable for colorectal cancer.

Table 11 displays the release kinetic parameters for numerous nanofiber mats. The diffusional release exponent varies in a range of 0.1112 to 0.3709 for all formulations that follow the Fickian diffusion mechanism, in which 5FU release is via diffusion or permeation through nanofiber matrices. The drug release period was 120 h, and the nanofiber degradation during this period was insignificant. Pectin-based nanofibers are also effective drug release matrices. Feng et al. [167] developed a polysaccharide-based nanofiber matrix for colon-targeted sustained release of salmon calcitonin (sCT), which is a peptide. Sodium alginate and sCT-loaded liposomes coated with pectin were the shell and core layers, respectively. The authors used different release models and found that the release followed a complex mechanism. Their core-shell nanofiber matrix further improved the burst release of sCT into simulated gastric and intestinal fluids.

Four mathematical models were used to examine the release kinetics of sCT contained in a core-shell nanofiber matrix dispersed in various media. The best model for each simulated gastrointestinal fluid was determined by the highest value of the coefficient of determination (*R*^2^). Table 12 shows the equations and calculated *R*^2^ values for each release rate measurement in various simulated media. The Higuchi has the highest *R*^2^ values for SGF and SIF media, indicating that Fickian diffusion mode was used for the release of sCT. The model’s best fit for the release in the SCF medium was a *b* value between 0.75 and 1, which indicates that the release of sCT in SCF followed a case II transportation (zero order) in which an additional mechanism must also be working in tandem with the zero order process. The authors concluded that the core-shell nanofiber mat is an appropriate delivery vehicle for sCT with a tunable, complex release process with different mechanisms.

In a very recent work [168], the electrospinning of the chitosan/pectin blend system was demonstrated using starch-derived cyclodextrin (hydroxypropyl-γ-cyclodextrin (HPγCD)) molecules. The nanofiber films having different chitosan/pectin ratios had high swelling (water uptake) rates, indicating hydrogel-forming capability. The authors used HPγCD molecules to encapsulate curcumin, with ∼89% curcumin loading efficiency. Their nanofibrous films demonstrated a pH-responsive release profile of curcumin in pH 5.4 and 7.4 media. The samples were labeled, for instance, as chitosan (3%)/pectin (1%) and chitosan (2%)/pectin (2%) systems with 30% (*w*/*v*) curcumin in CD (CD–Cur-IC) [168]. The release behavior was examined using five different kinetic models (zero-order, first-order, Higuchi, Korsmeyer-Peppas, and Hixson-Crowell). The applied formulations and the *R*^2^ values are summarized in Table 13. The highest *R*^2^ values (0.9485) were obtained for 3%Chit/30%CD–Cur-IC at pH 5.4, confirming a sustained release process compared to other release systems. A relatively higher *R*^2^ value was obtained with the Korsmeyer-Peppas model for 2%Chit/2%Pect/30%CD–Cur-IC at pH 5.4, which might indicate the existence of erosion-induced release, although the *R*^2^ value itself does not justify the superiority of one release process over the other. The authors concluded that the curcumin release from nanofiber mats has a pH-responsive, controlled release potential for normal skin and wounds.

Similarly, Cephalexin-incorporated electrospun core-shell nanofibers were made from poly(vinyl alcohol) (PVA) compounded with numerous polysaccharides such as chitosan (CH), carboxymethyl cellulose (CMC), carboxymethyl starch (CMS), and hydroxyproyl cellulose (HPC), with the PVA:polysaccharide ratio maintained at 90:10 [169]. The in vitro drug release experiments were fitted with several models to determine the drug release modes, including non-linear regression methods. Drug release was sluggish within the first 8 h. Furthermore, drug release was predominantly controlled by a diffusion mechanism, which makes this system suitable for wound repair. The kinetic models tested in this study were Korsmeyer-Peppas, Peppas-Sahlin, and Weibull. Applying the Korsmeyer-Peppas model for all the samples yielded *n* values lower than 0.5, indicating a Fickian drug diffusion or quasi-Fickian diffusion, and the transport of the drug occurred via the polymer network, not through the penetration of solvent or liquid into the nanofiber mats [169].

**Figure 6:**
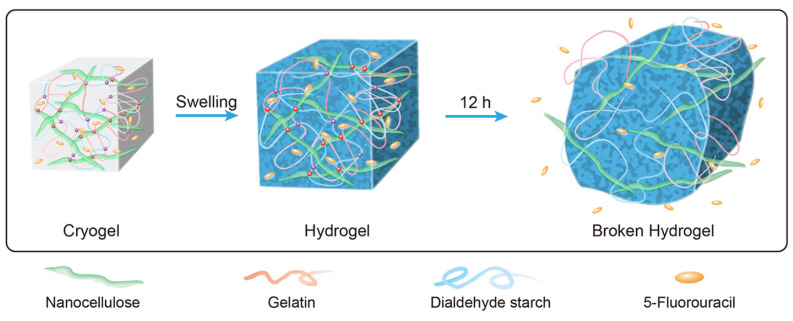
Nanocellulose/gelatin composite cryogels with controllable porosity, tunable swelling ratio, and reversible cross-linking structure were designed and used as the carriers for sustained drug release [170].

The mechanistic impact of manufacturing parameters and the release medium on drug release is exemplified in [170]. Nanocellulose/gelatin composite cryogels with tunable porosity, reversible nanofiber network features, and biocompatibility were developed by using chemical cross-linking of dialdehyde starch to enable controlled 5-fluorouracil (5-FU) release, as shown in Figure 6 [170]. Therein, the mode of drug release depended on the cryogel morphology and other parameters such as the NFC/gelling ratio, density, degree of cross-linking, and *pH* value. The pore structure could be tuned by modifying the relative concentrations of nanofibrous cellulose (NFC), gelatin, and dialdehyde starch. The mode of drug release depended on the cryogel morphology (NFC/gelling ratio, density and degree of cross-linking, pH value, etc.). The sustained release from the nanofiber networks could reach 12 h in a simulated intestinal environment [170]. The drug release data of three representative samples was fitted using zero-order, first-order, Higuchi, and Korsmeyer-Peppas models. Most of their nanofiber networks were more consistent with the Korsmeyer-Peppas model, which originates from Fick’s law theory and is an ideal drug release kinetic model. In another study, core-shell electrospun nanofibers were prepared by blending Dextran (Dex) and polyvinylpyrrolidone (PVP) in water dispersions. Afterwards, ciprofloxacin-loaded PVP/Dex nanofibers were made by the emulsion electrospinning method [171].

Emulsions were formed by dissolving the antibiotic in an edible oil. The authors applied Korsmeyer-Peppas, Peppas-Sahlin, and Weibull models to the experimental data to investigate the drug release mechanism from different forms of core-shell and polymer blend nanofibers. Release from all the samples studied was accurately described by the Weibull model. This indicated the occurrence of a release in accordance with Fickian diffusion in fractal space. They also argued that such a release mechanism indicates the dispersed drug did not percolate within the nanofiber network.

**Figure 7:**
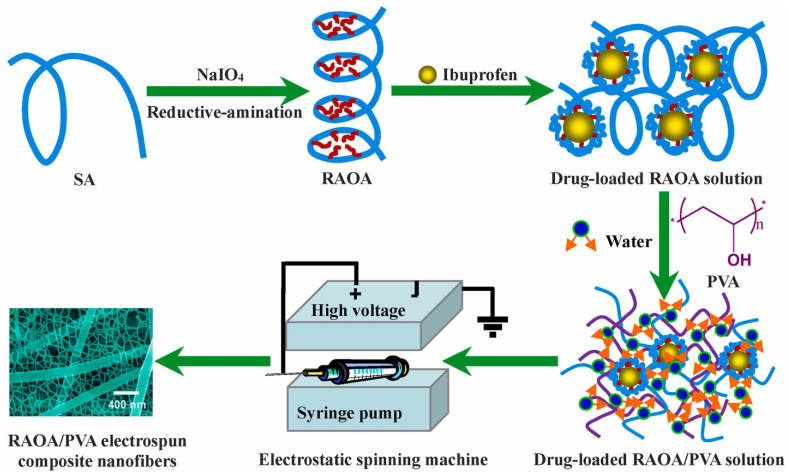
Schematic diagram of fabrication of drug-loaded RAOA/PVA electrospun composite nanofibers. Reductive-amination of oxidized alginate derivative (RAOA) through chemical modification can be electrospun into nanofiber mats and used for controlled release [172].

The RAOA destroyed the intramolecular hydrogen bonds in alginate, thus enhancing its molecular flexibility. Its chemical structure also allows the loading of hydrophobic drugs with negligible cytotoxicity into L929 cells. The loading of the hydrophobic anti-inflammatory drug, ibuprofen, with the aid of PVA is illustrated in Figure 7. Depending on the RAOA/PVA ratios in the solution blends, the encapsulation efficiency and the description of the release data with the Korsmeyer Peppas model showed efficiency exceeding 50% and non-Fickian diffusion, respectively. This result revealed that the swelling and degradation properties of the drug-encapsulating electrospun composite nanofibers and the diffusion of the drug jointly controlled the ibuprofen release rates.

Core-shell nanofibers were relatively unknown until recently, but now they are widely used in biomedicine thanks to their unique properties. They can be made either by electrospinning with a coaxial nozzle or by electrospinning immiscible polymer blends or emulsions through a single nozzle. The production of core-shell nanofibers with a wide range of compositions and diameters is made possible by a number of electrospinning parameters. Core-shell nanofibers have some advantages over monolithic nanofibers, including better drug, protein, gene, or probiotic amalgamation into the nanofibers, additional control over drug release, and maintenance of protein structure and activity during electrospinning [173]. It has been stated that the primary functions of core-shell fibers are to provide better preservation of sensitive bioactive molecules and superior control of biomolecule release. These functions have been expanded to include the ability to form fibers from almost any material and the ability to modify the physical and mechanical properties of the fibers in a flexible manner [174]. Moreover, core-shell nanofibers can allow the incorporation of dual drugs (one in each fiber) [175].

## 6. Drug Release from Polysaccharide-Based Nanoparticles

Polysaccharide-based nanoparticles find applications as nanoscale delivery systems, Pickering emulsion stabilizers, and material reinforcing additives in the fields of nanomedicine, cosmetics, and food [176]. In a recent work [177], starch nanoparticles from different botanical origin were prepared by nanoprecipitation. To deliver biodegradable antioxidant starch-quercetin nanoparticles, quercetin was incorporated into the starch nanoparticles. Researchers looked at how the source of the starch affected the release kinetics, antioxidant activity, and quercetin loading percentage. Along with the coefficient of determination *R*^2^ and the AIC, Table 14 shows the different release model parameters that the authors studied.

**Figure 8:**
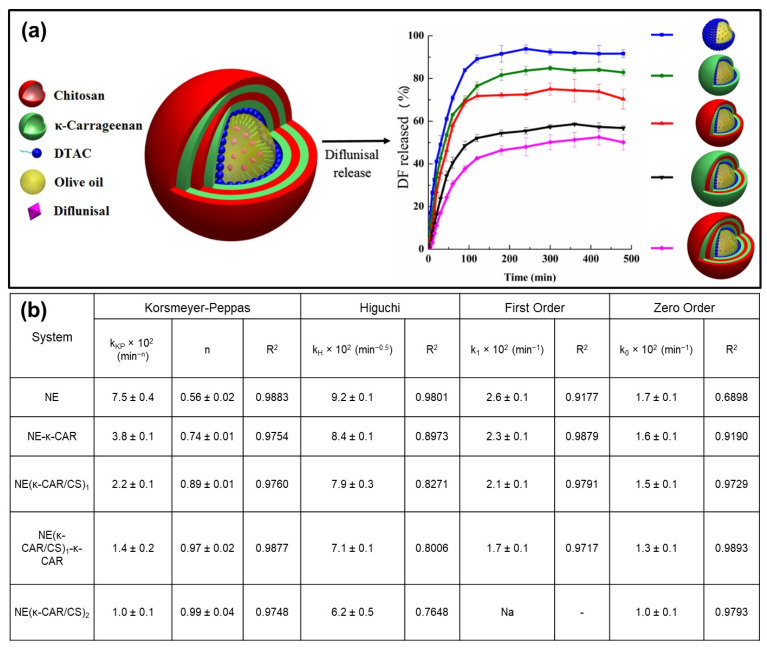
(**a**) Multilayer nanoparticles obtained from κ-carrageenan (κ-CAR) and chitosan (CS) were deposited onto olive oil nanoemulsion droplets (NE) via layer-by-layer (LbL) self-assembly. (**b**) Drug release models applied to the release measurements. Note that the notation NE(κ-CAR/CS)_x_ represents nanoemulsion as NE and x is the number of layers obtained from the NEs. Figure compiled with permission from [178], MDPI, 2018.

Note that the Akaike information criterion (AIC) is a mathematical method for assessing how well a model fits the data. In statistics, AIC is needed to compare different possible models and decide which one is the best fit for the data. They found that the Peppas-Sahlin, Weibull, and Korsmeyer-Peppas models fit their experimental data the best. The drug release from hydrophilic polymers with various geometries is defined by the Peppas-Sahlin model (Table 14). According to the model, the case-II relaxation mode and Fickian diffusion are two additive transport mechanisms that can have an impact on the release. For *M_t_*/*M*_∞_ < 0.6, the equation is valid. The first term in the equivalence gives the Fickian diffusion contribution, which results from the typical molecular drug diffusion caused by a chemical potential gradient. The second term denotes the contribution to case-II relaxation brought on by relaxation in hydrophilic polymers. This refers to polymer chain arrangements in water or biological fluids that are related to swelling. Table 14 demonstrates that for all of the starch-quercetin nanoparticles, the values of *k*_1_ (the Fickian diffusion constant) are orders of magnitude higher than *k*_2_ (the case II relaxation constant). This means the drug molecules are released mainly by Fickian diffusion. It has been argued that the negative *k*_1_ or *k*_2_ values can be interpreted as interference from the competing release component, but with a minor contribution to the final release profile [177]. There have also been reports of multilayer nanoparticles with the potential for drug release. Dodecyltrimethylammonium chloride (DTAC) was used to homogenize a nanoemulsion template to create multilayer nanocapsules, as reported in [178]. Because carrageenan sulfate groups can interact electrostatically with chitosan protonated amino groups to form polyelectrolyte nanocapsules, this method of synthesizing them was chosen (see Figure 8a). The authors looked at the performance of drug release using the lipophilic model drug diflunisal (DF). To define the primary release mechanisms as a function of the number of deposited layers and to categorize their applicability as controlled release systems, the results were fitted with various kinetic models. More specifically, the investigation results from the DF release mechanism from the oil-core nanoscale capsules were analyzed by relating them to the models shown in Figure 8b.

The *n* values for the NE(κ-CAR/CS)_1_---κ-CAR (three layers) and NE(κ-CAR/CS)_2_ (four layers) compounds are very close to 1 (see Table 9), signifying a Case II transport mode in the Korsmeyer-Peppas model. Although the results tabulated in Figure 8b (see also Figure 8a left panel) for the three-layer system display fitting statistics with *R*^2^ values around 0.98 for the zero-order model (a linear line profile), the visual inspection of the total % DF release curve of the 3-layer in Figure 8a deviates significantly from a zero-order behavior. The data in Figure 8a can be applied to such cases as Case II transport, which imitates the influence of polymer relaxation on molecules’ association in the matrix [178]. In a fascinating recent study, it was discovered how Triton X-100 and saponin, a naturally occurring surfactant derived from *Sapindus rarak*, affected the way that cellulose and starch nanoparticles were altered for the encapsulation and release of hydrophobic drugs [179]. The effectiveness of drug loading (Paclitaxel, PTX) was reported by the authors to have decreased. The authors have also reported that drug loading efficiency increased for both nanoparticles when 10 mM NaCl salt was added with the surfactant Triton X-100, and that Triton X-100 concentrations above 5 mM decreased dug encapsulation efficiency. In the case of saponin surfactant, there was no effect of salt addition on drug encapsulation efficiency. The use of saponin (100 ppm) with or without NaCl caused almost a triple increase in drug loading efficiency in the nanoparticles. The Higuchi model did not fit their data well, and hence they concluded that the release mechanism was not rapid dissolution of the drug as suggested by this model but rather diffusion of the dissolved drug. This means that upon contact with the release medium, as the matrix swells, the drug dissolves not rapidly, leaving the matrix, but rather starts diffusing in a much slower manner. The authors also indicated that the Korsmeyer-Peppas model failed to fit the overall experimental data at pH 5.8 and 7.4. They argued that the Korsmeyer Peppas is a semi-empirical power law that should correlate to either Fickian diffusion or a Case-II transport. The exponents, *n*, and the *R*^2^ values were below 0.5 and 0.9, respectively, and they indicated that since the *n* values were all below 0.5, the drug release should follow Fickian diffusion [179]. A sigmoidal model also fitted their data. The sigmoidal model can describe release profiles following an initial burst release of the drug and a slower sustained release over longer periods.

A recent study demonstrated the synthesis of green banana starch nanoparticles cross-linked with citric acid and encapsulating β-carotene [180]. Mean particle size, encapsulation efficiency, and β-carotene release in simulated gastric and intestinal fluids were measured and studied. The applied drug release models exhibited a diffusion of released β-carotene into food simulant media. In conclusion, the authors indicated that the cross-linked nanoparticles revealed better controlled release under gastric conditions, mainly in the simulated intestinal fluid, signifying that they can be suitable vehicles for intestine-specific targeting. The authors used the Kopcha model described by the relation *M*_t_ = *A*t^0.5^ + *B*t where *A* and *B* refer to the diffusion and erosion constants, respectively [180]. More specifically, in the Kopcha model, *A* and *B* are constants that signify the diffusion and erosion mechanisms involved in the molecule release from the starch nanoparticles. Some nanoparticles (depending on their crosslinking degree) showed greater *A* than *B* values in the release media, while the *A*/*B* ratio was greater than 1 in all cases, indicating that Fickian diffusion is the main mode of β-carotene release in all simulated media.

Capsaicin (CAP) is an alkaloid with multiple physiological effects, but its application as an encapsulated drug is challenging. Tao et al. [181] used capsaicin-loaded indica rice starch nanoparticles (CAP-IRSNPs) to enable controlled release from the matrices with average particle sizes of 617.84 ± 6.38 nm, an encapsulation efficiency of 70.05 ± 1.78% and a loading capacity of 13.41 ± 0.18%. They tested numerous theoretical release models, i.e., zero-order kinetics model, first-order kinetics model, Higuchi, Korsmeyer-Peppas, and Hixson-Crowell equations and models, to elucidate drug release mechanisms, as shown in Table 15.

The best expressive kinetics model was chosen for the experimental measurements based on the results shown in Table 15 by comparing the coefficient of determination (*R*^2^). The first-order and Korsmeyer-Peppas models with *R*^2^ greater than 0.9 best fit the data. The highest *R*^2^ value for the first-order model indicates that the drug release rate variations and kinetics were affected by capsaicin concentration. Furthermore, the capsaicin release profiles of two composites were second-best fitted with the Korsmeyer-Peppas model, with *n* values for the release exponent of 0.032 and 0.366, respectively. The values were less than 0.45, indicating that capsaicin release from IRSNP matrices follows Fickian and/or molecular diffusion of drug chemical potential gradients [181].

Ergin et al. [182] developed a colon-targeted delivery system for enhancing the oral bioavailability of S-adenosyl-l-methionine (SAMe). They prepared nanoparticle-in-microparticle (NIM) formulations containing SAMe using pectin. The physicochemical properties of nanoparticles were studied, while an optimization study was carried out by factorial design to understand the effect of formulation variables on nanoparticle properties. The drug release models, shown in Table 16, were fitted to the drug release data. The drug release data after 2 h in a simulated colon fluid were used for NIMs since they all had delayed release structures. Results were statistically evaluated by using determination coefficients (*R*^2^), adjusted determination coefficients (*R*^2^_adjusted_), Akaike Information Criteria (AIC), and Model Selection Criteria (MSC). Release constants (*k*) and *n* values were estimated for each model used (Table 16). The AIC method comprises the sum of squared mean differences between measured and calculated values pertaining to the selected method. A lower AIC value indicates that the model is better. The MSC model is a modified reciprocal form of the AIC. MSC values greater than 2 or 3 yield information about the aptness of the model [182]. The evaluation was based on the highest *R*^2^, *R*^2^_adjusted_, MSC, and AIC values.

According to Table 16, the Korsmeyer-Peppas model appears to be the best fit to the release data, with the Higuchi model coming in second. Korsmeyer-Peppas parameter *n* was 0.43, indicating the Fickian diffusion mechanism for NIMs. The *n* value for nanoparticles was 0.376. Although the Fickian diffusion mode is thought to be valid within 0.43 and 0.50, it can be defined by *n* values less than 0.43, i.e., around 0.3 ± 0.1, for drug release from spherical polymer particles with a wide size distribution, as stated in [181,182,183]. The Higuchi model revealed a diffusion-controlled release mechanism as a variant of Korsmeyer-Peppas (*n* = 12).

**Figure 9:**
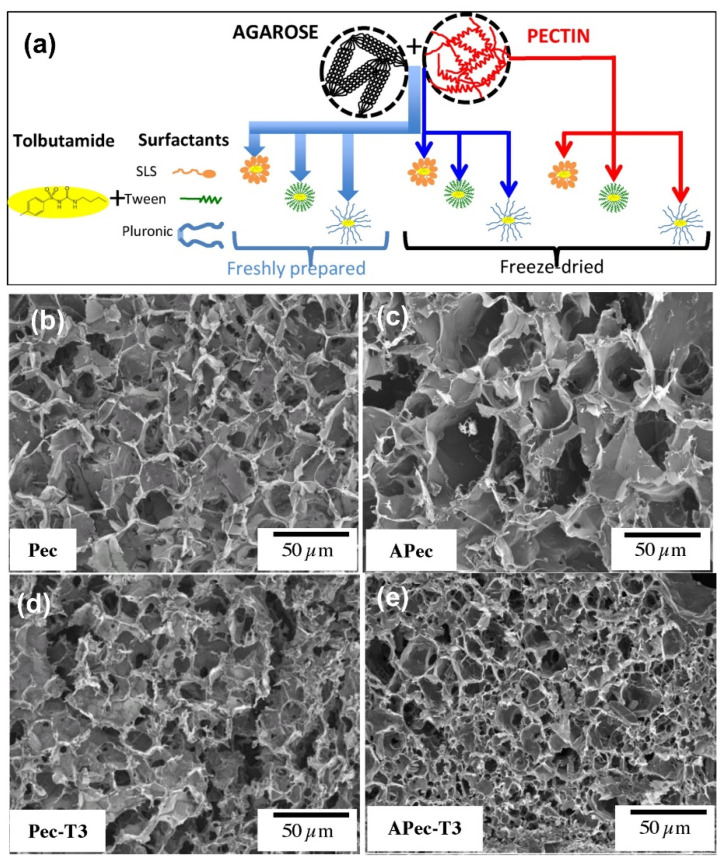
(**a**) A schematic representation of nanoscale hydrogels and pore architecture as well as used surfactants and freeze-drying and oral drug delivery kinetics. (**b**–**e**) SEM micrographs of freeze-dried pectin and blend systems. The samples concretions are: Pec: 30 mg Pec and 10 mg drug, APec: 15 mg Agarose, 15 mg. Pec and 10 mg drug. Pec-T3 30 mg Pec, 30 mg Tween 10 mg drug APec-T3: 15 mg Agarose, 15 mg. Pec, 30 mg Tween and 10 mg drug [184].

Pectin-based, resistant, interactive, and versatile hydrogel nanoparticles for oral administration have also been prepared [184]. A number of surfactants were used for drug inclusion, such as Pluronic, Tween, and Na Lauryl Sulfate, which the authors indicated may modulate the drug release patterns. As a model drug, tolbutamide was chosen. It shows a discrete and pH-dependent solubility in water. To manipulate the morphology of the nanoparticle gels, blending with agarose or freeze-drying was employed (see Figure 9a). Tolbutamide release kinetics from freshly prepared matrices were fitted by the Higuchi model, whereas the lyophilized ones followed the Korsmeyer-Peppas model. Therefore, the nanoparticle-hydrogel chain rearrangement process can tune the release during the rehydration process. The examination of the morphology of the prepared nanoparticle/hydrogel systems indicated a honeycomb-like structure, the size and density of which were dependent on the blend system concentrations, surfactant type, and fresh preparation or freeze-drying, as shown in Figure 9b–e. For instance, a sample that contained 15 mg of agarose, 15 mg of pectin, 30 mg of Tween, and 10 mg of the drug in Figure 9e had a much finer and denser porous structure. Drug release data indicated that the release from freeze-dried matrices started with rehydration, governed by the microstructure, but also with subsequent drug dissolution and diffusion processes. In the case of freshly prepared samples, the drug could diffuse from the already highly hydrated hydrogel to the surrounding medium, following the Higuchi model [184]. Drug release from the freeze-dried samples fitted well to the Kosmeyer-Peppas and confirmed that the release from these matrices was governed by the relaxing and rearrangement processes that disrupt the hydrogel chains during rehydration.

Due to the pH sensitivity of chitosan, Aydin and Pulat [185] reported 5-fluorouracil (5-FU) encapsulated chitosan nanoparticles to investigate the potential of localized drug delivery for tumor locations. Chitosan nanoparticles were approximately 200 nm in size. Their findings revealed a significant swelling response for pH 5 particles with a final particle diameter of 450 nm. After 408 h of incubation, in vitro release studies revealed a controlled and sustained release of 5-FU from the nanoparticles, with cumulative release values ranging from 29.1–60.8% due to different pH environments.

The zero order, first order, Higuchi, Hixson-Crowell, Korsmeyer-Peppas, and Kopcha models were used to assess the kinetics of 5-FU release from nanoparticles. The determination coefficient values (*R*^2^) and release parameters extracted from the model fitting to the measured data are shown in Table 17. Based on determination coefficient values, the release data was best described by the Higuchi model at all pH values, indicating that 5FU is released by diffusion, as shown in Table 17. Furthermore, the exponent, n, of the Korsmeyer-Peppas release model (high correlation values) is around 0.3, confirming that Fickian diffusion is the controlling factor in drug release. Because the Kopcha model can predict the contribution due to diffusion and polymer relaxation, the results in Table 17 confirmed that drug release occurred via Fickian diffusion.

It is critical to understand the precise mass transport mechanisms involved in the drug release and to forecast the subsequent drug release kinetics in order to evaluate a given drug release mode in a nanoscale polysaccharide therapeutic system. As we have seen, numerous mathematical models have been created to design a variety of straightforward and intricate drug delivery systems as well as to predict the overall performance of the release. Once polysaccharide-derived drug release materials are commercialized, it will be very important to distinguish between how to apply these equations to recognize the various factors that affect the release velocity and how the dissolution performances can vary and affect the efficacy or the therapeutic regimen of patients [186,187,188,189,190,191].

**Figure 10:**
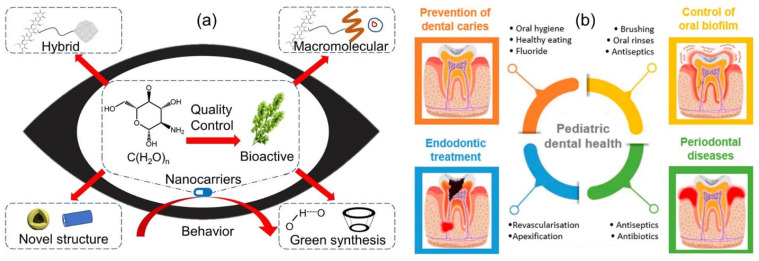
(**a**) Future development routes of polysaccharide-based nanomaterials for ocular drug delivery [190]. No written permission required MDPI Publishing, Basel Switzerland (**b**) The most common diseases and oral conditions associated with pediatric dental patients [193].

For example, in recent years, polysaccharide-based nanomaterials have been extensively investigated as ideal carriers to enhance drug bioavailability in the ocular system due to their biocompatibility and drug encapsulation [191]. Indeed, many recent studies have focused on the structural instability of polysaccharides in the synthesis of nanocarriers and their translations, including bioactive polysaccharide-based nanomaterials, new strategies in nanocarrier design, and their behavior in ophthalmic therapy. The focus is on possibilities (see Figure 10a). Additionally, compared to most synthetic polymers, their low immunogenicity and low toxicity provide an advantage for the development of pediatric formulations [190]. In-depth research on the safety and effectiveness of nanoscale innovative therapeutics is required before they can be introduced into pediatric dental practice. However, polysaccharide-based drug delivery matrices offer promising therapeutic options for the management of the most prevalent dental diseases and conditions in children, including periodontitis, oral biofilm prevention, endodontic therapy, and the prevention of dental caries [192,193] (see Figure 10b).

Kurczewska [194] indicates that even though there is a large diversity of formulations in polysaccharide-based drug delivery systems, the most widely used materials are alginate, chitosan, hyaluronic acid, pectin, dextran, starch, and cellulose.

## 7. Pharmacological Activity of Polysaccharides and Their Stimulus Release Properties

In addition to being excellent vehicles for drug loading and controlled release, many polysaccharides have very advantageous pharmacological properties [195]. Polysaccharides are isolated from natural resources such as plants, animals, fungi, and seaweed and have received increased attention in recent years due to their wide range of pharmacological activities, including anticancer, immunomodulation, antioxidant, antiseptic, and anti-inflammatory effects [196]. Particularly, it has been demonstrated that sulfated polysaccharides from seaweed, arabinogalactan, galactomannan, and pectic polysaccharides derived from higher plants, β-glucans and glycoproteins derived from mushrooms, and pectic polysaccharides derived from higher plants all possess antioxidant and immunomodulatory activities [197]. The (1→6)-α-d-glucan from *Dimocarpus longan* is thought to have excellent antitumor properties, and the (1→3)-β-d-glucan is well-known for its ability to regulate the immune system [198]. Recent research has revealed that *Bullacta exarata*’s mannoglucan and sulfated polysaccharide extracts have a variety of biological benefits, including hepatoprotective, antioxidant, anticancer, antihypertension, and hypocholesterolemic effects [199]. According to topical pharmacological research, the polysaccharides isolated from *Cordyceps sinensis* have anti-fibrosis, anti-tumor, and immunomodulatory properties [200]. *Sargassum pallidum* polysaccharides have been shown in modern pharmacological studies to have a wide range of beneficial health-promoting properties, including antioxidant, anticancer, hypolipidemic, and immunomodulatory activities [201].

Very recent literature reviews also highlighted the importance of stimulus-based release from polysaccharides [202,203]. Li et al. [202] reviewed combined cancer immunotherapy advancements in polysaccharide-based stimulus-responsive nanomedicines. System development, targeted delivery, drug release, and improved antitumor effects were tabulated and discussed, with particular emphasis on precise and controlled drug release in response to internal or external stimulus that was tailored towards cancer immunotherapy. For instance, pH-responsive starch-based hydrogels produced by graft copolymerizing with various acrylic monomers could be designed to swell in a controlled manner. This would regulate, for instance, how quickly caffeine (a model drug) was released [204]. Additionally, an authoritative review by Alvarez-Lorenzo et al. [205] presented ionic polysaccharides that can be crosslinked to form hydrogel networks that are sensitive to a variety of internal and external stimuli, making them ideal for switching drug release on and off via various mechanisms. They examined the current state of the art in crosslinked ionic polysaccharides as components of drug delivery systems that can regulate drug release in response to changes in pH, ion nature and concentration, electric and magnetic field intensity, light wavelength, temperature, redox potential, and specific molecules (enzymes, illness markers, and so on).

Polysaccharides such as chitosan, chondroitin sulfate, dextrans, cyclodextrins, guar gum, pectin, inulin, and others have been utilized for colon-specific delivery. The readers are referred to Tiwari et al. [206], who summarized various polysaccharides and approaches for stimuli-responsive drug delivery for colon targeting, with a focus on drug delivery systems such as microspheres, nanoparticles, hydrogels, matrices, and beads. Finally, it is important to state that in vivo and ex vivo studies on various polysaccharides are of immense importance. For instance, a potentially useful biomedical substance is bacterial nanocellulose (BNC). Three-dimensional (3D) BNC biomaterials’ hemocompatibility (haemolysis and thrombogenicity) as well as acute and subchronic immune reactions were examined in [207]. Based on these in vivo studies, it was reported that 3D BNC only induced a mild acute inflammatory response, not a foreign body or chronic inflammatory response, and did not affect the wound’s ability to heal [207]. Another study [208] developed a hydrogel based on hyaluronic acid (HA), methylcellulose (MC), and Poloxamer Pluronic-F127 (F127). It was developed as an endoscopic vehicle for delivering biological drugs with proven efficacy in acute and chronic EC in rats while causing less immunogenicity. Similarly, in [209], it was discussed how to use in situ-forming pectin hydrogels as cell carriers for tissue engineering and regenerative medicine strategies. To promote cell-matrix adhesion, pectin was successfully grafted with a peptide. For at least 14 days, human mesenchymal stem cells (hMSCs) embedded in pectin hydrogels were viable and metabolically active. Furthermore, they formed intercellular networks within the pectin hydrogels, migrated outward, and produced endogenous extracellular matrix. Polysaccharide-based hydrogels can be designed with varying biodegradability under physiological conditions, depending on the application. In fact, this property is directly related to their in vivo biocompatibility [210]. The ability to degrade slowly or quickly is determined by the polysaccharide used, the chemical nature of the linker, the degree of crosslinking, and the type of further functionalization with an additional ligand [210].

## 8. Biopharmaceutics and Pharmacokinetics Considerations

A key area of the pharmaceutical sciences is known as "biopharmaceutics," which studies the relationship between a drug’s physicochemical properties in dosage form and the pharmacology, toxicology, or clinical response that needs to be studied after the drug is administered. The dosing regimen affects both the safety and effectiveness of a drug. For numerous medications, there can be significant variations in the ideal dosage and dosing intervals. Additionally, the ideal dosage for a single drug can vary greatly between patients [211]. The processes of the drug’s absorption, distribution, metabolism, and excretion (ADME) pattern are all included in pharmacokinetics, which is the study of the time course of a drug within the body (the amount and duration of systemic exposure to the drug). Pharmacokinetic parameters are typically derived from the analysis of drug concentrations in plasma or blood [212]. The key pharmacokinetic variables that characterize the ADME processes are given as the area under the concentration-time curve (AUC), the volume of distribution (*V*_d_), the half-life (*t*_1/2_), and the clearance (*C*l) of the drug. They also include the maximum plasma concentration and the time at which this concentration is reached (*C*_max_ and *T*_max_, respectively), as well as the area under the concentration-time curve (AUC) [213]. Due to their unusual chemical and physical properties, nano-engineered matrices for the delivery of cytotoxic agents in particular have significantly altered the pharmacokinetic behavior of the parent drugs. Because the physicochemistry of nanocarriers is so important, it is imperative to briefly discuss here the key factors that affect their kinetics [214].

When used in conjunction with pharmacodynamic modeling, physiologically based pharmacokinetic (PBPK) modeling has proven to be a useful instrument for characterizing and predicting the systemic disposition, target exposure, efficacy, and toxicity of various types of drugs [215]. Although it is frequently touted as a benefit, the multi-functionality of nanoscale drug matrices also leads to different and more complex in vivo disposition properties when compared to a conventional formulation of the same drugs. For instance, tissue partition coefficients, cell uptake rate, and drug release rate are likely to be time- and tissue-dependent, making it difficult to parameterize their spatiotemporal disposition in PBPK models. Meanwhile, due to differences in biological conditioning, model parameters calibrated against one administration route may not predict the outcome of another. Nanocarriers are a diverse population of individual particles with varying particle properties. The average particle properties may not accurately predict the behavior of individual nanocarriers. Two formulations with the same average but different property distributions can have vastly different disposition performances. As such, there is a lack of a reliable analytical method for characterizing and tracking nanoparticles as individual nanoparticles in vitro and in vivo [216]. Unsolved issues like incomplete knowledge of nanocarrier elimination in vivo, potential toxicity, and unaccounted-for pharmacokinetic behavior prevent nanocarriers from being used clinically for drug delivery. Hence, it is important to focus not only on drug delivery at the nanoscale but also on developing stable, low-toxicity, clinically applicable nanoscale frameworks for drug delivery [217].

## 9. Statistical Analysis of Release Profiles

In general, mathematical models are used as a perception of a real system with assumptions and simplifications and are able to interpret and predict experimental results. These mathematical expressions, which include additional parameters in their equation that may or may not be related to physicochemical properties, provide a qualitative and quantitative description of the primary phenomena involved in drug release. The models include mechanistic realistic systems in which real phenomena like drug diffusion or dissolution, erosion, swelling, precipitation, and polymer degradation are taken into account, making them complex to apply. Empirical or semi-empirical theories are not based on actual chemical, physical, or biological phenomena, but they are somewhat realistic from a physical-chemical standpoint, making them generally simple to apply and explain [218].

Simple linear regression analysis using the sum of squares method is generally used with the help of software such as the free “*R* Project for Statistical Computing” to fit each model. Such software can be used to calculate the model acceptance criteria, including the linear regression indexes (intercept I and slope S), regression coefficient (*R*^2^), adjusted or corrected regression coefficient (*R*^2^_a_), sum of squares of residual (SSR), sum of squares of error (SSE), sum square of total variation (SST), T-statistic, and F-statistic. The percentage of the variation in the dependent variable that can be predicted from the independent variable or variables is known as the coefficient of determination, abbreviated *R*^2^ or *r*^2^, in statistics. The adjusted *R*^2^ parameter attempts to account for the phenomenon of *R*^2^ increasing automatically when additional explanatory variables are added to the model [219]. There are numerous methods for adjusting. The adjusted *R*^2^_a_ can be negative, but it must always be less than or equal to *R*^2^. Unlike *R*^2^, adjusted *R*^2^_a_ increases only when the increase in *R*^2^ (due to the addition of a new explanatory variable) is greater than would be expected by chance [219]. The Akaike information criterion (AIC) is a widely used tool in statistical modeling [220]. AIC was introduced as an extension to the maximum likelihood principle, making it the first model selection criterion to gain widespread acceptance. Once the structure and dimensions of a model have been defined, maximum likelihood is typically used to estimate its parameters.

Another statistical criterion for model selection that is gaining popularity in the field of dissolution data modeling is the model selection criterion, or MSC [221]. The MSC has been normalized to be independent of the scaling of the data points and is a modified reciprocal form of the AIC. The model with the largest MSC will be the most appropriate model when comparing various models. As a result, getting a sense of what the MSC means in terms of how well the model fits the data is relatively simple. A MSC value of two to three or higher typically indicates a good fit [221,222]. Readers who are interested in an in-depth analysis of model comparison algorithms for drug release can consult the work of Zhang et al. [221], which also provides free software for drug release data analysis.

## 10. Summary and Future Trends

There has been a lot of effort put into the development of biodegradable and biocompatible nanomaterials that possess several potential benefits, such as passive or active targeting. Additionally, it is important to note that the mucoadhesive properties of polysaccharides are very important for ensuring delivery systems remain at the site of absorption over an extended period of time. In this study, we reviewed a number of important nanoscale polysaccharide systems in terms of their application challenges, strengths, and weaknesses as drug delivery systems, with particular attention to drug release kinetics [223]. Polysaccharides can be classified into two main clusters: polyelectrolytes and non-polyelectrolytes. Polyelectrolytes can be further divided into cationic (chitosan), anionic (alginate, heparin, pectin, hyaluronic acid), and neutral (pullulan, dextran) subgroups based on their intrinsic charge. In particular, the use of pectin and its derivatives with low water solubility deserves more attention. This property of pectin can be either an advantage or a disadvantage, depending on the active ingredient content.

The classification of polysaccharide-based nanoparticles developed as drug carriers and delivery systems is presented in Table 18.

On the other hand, mathematical modeling of drug release from nanoscale polysaccharide matrices is of great importance, with particular attention to describing the chemical and physical phenomena that govern drug delivery. The concept of personalized medicine proposes treating each individual patient as a unique subject through optimized medication. The use of nanoscale polysaccharides in personalized medicine can reduce side effects and enhance the efficacy of the therapeutic treatment. Combining patient uniqueness with nanoscale engineering of drug delivery systems based on polysaccharides requires careful attention to drug release kinetics and the models that describe such dynamics. Mathematical drug release models can unravel what is not directly observable, such as molecules, atoms, and subatomic particles, and help establish certain standards that can be specifically applied to a certain class of patients [236].

Nanoscale polysaccharide drug delivery vehicles can stay in the blood circulatory system for a sustained period and enable the release of integrated drugs as per the specified dose. Hence, fewer plasma fluctuations with reduced adverse effects may be expected. Being natural and non-toxic, they penetrate into the tissue while facilitating easy uptake of the drug by cells, allowing efficient drug delivery, and causing the desired effect at the targeted location. Their polyelectrolyte features can enable direct interactions to treat the diseased cells with improved efficiency and reduced or negligible side effects. Many new drug release materials are being engineered from nanoscale polysaccharides in order to more effectively deliver promising new drugs to target tissues and cells. Many newly developed nanoparticles made from this type of material also demonstrated innovative methods for getting oral biologics past the intestinal barrier—something that has been elusive for generations of pharmaceutical designers [235,237].

Mathematical drug release models from nanoscale matrices have advanced our understanding of cellular transport mechanisms, and many more research groups and reports have used these insights to develop potential new drugs encapsulated in polysaccharides. Design, characterization, analysis, formulation, and delivery should be optimized. As therapeutic modalities have expanded beyond small molecules to include nucleic acids, peptides, proteins, and antibodies, nanoscale polysaccharide drug matrices have emerged to address challenges such as live-cell delivery with nano-engineered polysaccharides [237]. These matrices must be designed to mimic key biological processes like host-responsive insulin secretion and must be used more in advanced cell therapies to reduce dosing frequency and minimize medical interventions. Cell therapies with polysaccharides should utilize established methods to modify drugs and their microenvironment to control drug action, efficacy, and toxicity; equally, specific improvements demonstrated with polysaccharide-cell methodologies can also support other classes of therapeutics.

Moreover, nanoparticles fabricated from polysaccharides are becoming more common platforms to facilitate sustained release and imaging and are thus becoming materials of choice in nano-theranostics. To date, however, nanoscale polysaccharide sustained drug release and modeling are still at the academic research level. The transformation of some of this research into clinical trials must start even though substantial work is still required in reliable manufacturing and scale-up, the establishment of regulatory means for therapy and diagnostics, and eventual theranostic innovations like advanced multimodal imaging [237].

## Figures and Tables

**Table 1:** Common polysaccharides and their origin.

Classification of Polysaccharides Based on Different Natural Sources			
Higher Plants	Algal	Animal Origin	Microbial
Starch	Alginates	Chitin	Dextran
Cellulose	Galactans	Chitosan	Gellan gum
Guar gum	Carrageenan	Glycosaminoglycans	Pullulan
Gum Arabic	Fucoidan	Hyaluronic acid	Xanthan gum
Locust bean gum	Ulvan (green macroalgae)		

**Table 2:** Polysaccharides as drugs and their biological and targeted applications. Data reproduced with permission from [27], Elsevier 2022.

Source	Polysaccharide	Drug Form	Biological Activity and Applications
Animal	Heparin	Heparin sodium cream; heparin sodium lozenge; low molecular weight heparin sodium gel; heparin calcium for injection; heparin (sodium, calcium) injection	Anticoagulant, antiviral [29], a biosensor for thrombin [30], stabilize, deliver, and enhance growth factors like FGF-2 [31], anti-inflammatory and anti-angiogenic activity [32]
	Chondroitin sulfate	Chondroitin sulfate tablets; chondroitin sulfate (chondroitin sulfate A sodium) capsules; chondroitin sulfate (chondroitin sulfate A sodium) injection	Coatings [31], cell growth, differentiation, morphogenesis, cell migration, and bacterial/viral infections [33], interactions with matrix proteins, activation of growth factors, regulation of angiogenesis, and melanoma cell invasion and proliferation [34], osteoarthritis [35]
	Hyaluronic acid	Sodium hyaluronate injection; sodium hyaluronate eye drops	Drug carriers [36], anti-arthritic [37], osteoarthritis [38]
Plant	Astragalus PS	Astragalus polysaccharide injection (2-(chloromethyl)-4-(4-nitrophenyl)-1,3-thiazole)	Immunoregulatory [39], anti-oxidative [40], antiviral [41], and anti-tumor [42,43]
	Ginseng PS	Ginseng polysaccharide injections	Immunostimulant [44], hypoglycemic [45], anti-inflammatory [46]
	Fucoidan PS	Active pharmaceutical ingredient	Cell proliferation and differentiation [47], immune modulation, cancer inhibition, and pathogen inhibition [48], antioxidant [49], antitumor [50], antiviral [51]
Microbial	Lentinan PS	Lentinan injection; lentinan capsules; lentinus edodes mycelia polysaccharides tablets	Immunologic activities [52], antitumor [53], Hepatoprotective, and Antiviral [54]
	Poria PS	Poria polysaccharide oral solution capsular	Antitumor [55], immunomodulation, anti-inflammation, antioxidation, anti-aging, antihepatitic, antidiabetics, and anti-hemorrhagic fever [56]
	Capsular PS	Vi polysaccharides typhoid vaccine; pneumococcal vaccine polyvalent; group A and C meningococcal polysaccharide vaccine	Vaccines and passive antibody therapies [57]
	Dextran	Dextran 40 glucose injection; dextran 70 eye drops; low molecule dextran	Biotechnological applications [58]

**Table 3:** Physical or chemical trigger-driven polysaccharide material systems and their potential drug delivery applications. Data compiled with permission from [61] under Attribution-NonCommercial 4.0 International (CC BY-NC 4.0) license, SAGE Publishers 2018.

Smart Response	Biopolymer	Blend	Application	Reference
Sol–gel transition	Kappa carrageenan	Gellan gum	Ocular safety	[62]
		Methylcellulose	Ophthalmic drug delivery system	[63]
	Alginate	Gelrite	Ocular safety	[64]
		Hydroxypropyl methyl cellulose	Ophthalmic drug delivery system	[65]
		–	Ophthalmic drug delivery system	[66]
		Aminocaproic acid	Drug delivery	[67]
	Dextran	Tyramine	Drug delivery/tissue engineering	[68]
	Hyaluronic acid	Tyramine	Drug delivery/tissue engineering	[69]
	Modified chitosan (chitosan-graft-glycolic acid)	–	Tissue engineering	[70]
Swelling	Modified chitosan (*N*-succinyl-chitosan)	Aldehyde hyaluronic acid	Tissue engineering	[71]
	Modified calmodulin (calcium-binding protein)	3-[2-(trifluoromethyl)-10H-phenothiazin-10-yl]propan-1-amine	Drug delivery/microfluidic	[72]
	Poly(l-glutamic acid)	Phloretic acid	3D cell culture and recovery/tissue engineering	[73]
Degradation and release	Poly(l-glutamic acid)	Phloretic acid	3D cell culture and recovery/tissue engineering	[73]
	Alginate	–	Drug delivery	[74]
	Dextran	–	Drug delivery	[75]
Self-assembly/folding	Peptide-hyaluronan hybrid hydrogel	–	Controlled release	[76]

**Table 4:** Commonly employed drug release models.

Model Name	Equation	Measurable Variables and Definitions
Zero order	$Q_{t}=Q_{0}-K_{0}t$	*Q_t_* = Cumulative amount of drug released at time *t*, *t*; *Q*_0_ = Initial drug amount in the matrix; *K*_0_ = zero-order release rate constant
First-order	$\begin{array}{c} \log Q_{t}=\log Q_{0}-\frac{K_{1}t}{3.303} \end{array}$	*Q_t_* = Cumulative amount of drug released at time *t*; *Q*_0_ = Initial amount of drug in the matrix; *K*_1_ = First-order release rate constant
Higuchi	$Q_{t}=K_{H}t^{0.5}$	*Q_t_* = Cumulative amount of drug released at time *t*; *K*_H_ = Higuchi’s release rate constant
Hixson-Crowell	$\begin{array}{l} Q_{0}^{1/3}-Q_{t}^{1/3}\\ =K_{S}t \end{array}$	*Q_t_* = Cumulative amount of drug released at time *t*; *Q*_0_ = Initial amount of drug in the matrix; *K_s_* = Release rate constant
Baker–Lonsdale	$\begin{array}{l} \frac{3}{2}[1-{\left(1-\frac{M_{t}}{M_{\alpha }}\right)}^{2/3}]\\ -\frac{M_{t}}{M_{\alpha }}=\frac{3D_{m}C_{ms}}{r_{0}^{2}C_{0}}t \end{array}$	*M_t_* = Amount of drug released at time *t*; *M_α_* = Amount of drug released at an initial time; *D_m_* = diffusion coefficient; *C_ms_* = drug solubility in the matrix; *r*_0_ = radius of the spherical matrix; *C*_0_ = initial concentration of drug in the matrix
Korsmeyer–Peppas	$\frac{M_{t}}{M_{\alpha }}=kt^{n}$	*M_t_*/*M_α_* = fraction of drug released at time *t*; *k* = kinetic constant; *n* = release exponent relating to transport mechanism
Hopfenberg	$\begin{array}{l} \frac{M_{t}}{M_{\alpha }}=1\\ -{\left[1-\frac{K_{0}}{C_{0}a_{0}}\right]}^{n} \end{array}$	*M_t_*/*M_α_* = fraction of drug dissolved; *K*_0_ = erosion rate constant; *C*_0_ = initial concentration of drug in the matrix; *a*_0_ = initial radius for matrix; *n* = 1, 2 and 3 for a slab, cylinder and sphere, respectively.
Poiseuille’s law of laminar flow	$\frac{dM}{dt}=\frac{\pi c}{8}\frac{r^{4}}{\eta }\frac{P_{1}-P_{2}}{h}$	d*M*/d*t* = drug release rate; *c* = concentration of drug in matrix; *r* = radius of orifice; *η* = viscosity of matrix; P_1_ − P_2_ = pressure difference between the inside and outside of the membrane.
Weibull	$\begin{array}{l} \log\left[-\ln\left(1-m\right)\right]\\ =b\log\left(t-T_{i}\right)\\ -\log a \end{array}$	*m* = fraction of the drug in solution at time *t*; *a* = time scale of the process; *b* = shape parameter; *T_i_* = lag time

**Table 5:** Some examples of drug loaded polysaccharide systems and their applications.

Drug	Mode of Release	Polysaccharide Matrix	Remarks	Reference
Dexamethasone and Levofloxacin	Opthalmic delivery	Glycol chitosan/hyalouranic acid hydrogel film	Burst release of levofloxacin followed by the sustained release for dexamethasone	[101]
Miconazole nitrate	Oral delivery	Chitosan-HPMC/Pectin film	Chitosan-HPMC film found to be superior as drug delivery support.	[102]
Peptides and proteins	Transdermal	Chitosan-tamarind seed polysaccharide composite film	The film is antimicrobial and stable.	[103]
Paracetamol	Colon delivery	Pectin/chitosan/hydroxyl propyl methyl cellulose films	Bimodal drug release	[104]
Bioactive materials	Wound dressing	Chitosan cyclodextrin inclusion complex based film	Presence of cyclodextrin prevent the loss of bioactives due to evaporation	[105]
Paracetyl aminophenol	In vitro	Silver loaded hydroxyl ethylacryl chitosan-sodium alginate hydrogel film	Presence of silver prolonged the drug release rate	[106]
Ketorolec methane	Transdermal delivery	Cellulose/nanofibril chitosan transdermal film	Sustained release of drug	[107]
Ellagic acid	Transdermal	Chitosan-ellagic acid-based films	Induce apoptotic death in human carcinoma cells.	[108]
Ciprofloxacin	In vitro	Chitosan/PVP/Guargum blended films	pH sensitive ternary blend film for the controlled release.	[109]
Betamethasone, Sulfadiazine	In vitro	Chitosan nanocellulose film	Ideal for wound dressings	[110]

**Table 6:** Properties of some common antibiotics Note that “cidal” means effectively killing bacteria. Data reproduced with permission from [112], Elsevier 2019.

Drug	Primary Effect	Spectrum	Side Effects
Ampicillin	Cidal	Broad (Gram+, some Gram−)	Allergic response, diarrhea, anemia
Bacitracin	Cidal	Narrow (Gram+)	Renal injury if injected
Carbenicillin	Cidal	Broad (Gram+, many Gram–)	Allergic responses, nausea, anemia
Cephalosporins	Cidal	Broad (Gram+, some Gram–)	Allergic responses, thrombophlebitis, renal injury
Chloramphenicol	Static	Broad (Gram+, Gram–; Rickettsia and Chlamydia)	Depressed bone marrow function, allergic reactions
Ciprofloxacin	Cidal	Broad (Gram+, Gram–)	Gastrointestinal upset, allergic responses
Clindamycin	Static	Narrow (Gram+, anaerobes)	Diarrhea
Dapsone	Static	Narrow (mycobacteria)	Anemia, allergic responses
Erythromycin	Static	Narrow (Gram+, mycoplasma)	Gastrointestinal upset, hepatic injury
Gentamicin	Cidal	Narrow (Gram–)	Allergic responses, nausea, loss of hearing, renal damage
Isoniazid	Static	Narrow (mycobacteria)	Allergic reactions, gastrointestinal upset, hepatic injury
Methicillin	Cidal	Narrow (Gram+)	Allergic responses, renal toxicity, anemia
Penicillin	Cidal	Narrow (Gram+)	Allergic responses, nausea, anemia
Polymyxin B	Cidal	Narrow (Gram–)	Renal damage, neurotoxic reactions
Rifampin	Static	Broad (Gram–, mycobacteria)	Hepatic injury, nausea, allergic responses
Streptomycin	Cidal	Broad (Gram+, Gram–; mycobacteria)	Allergic responses, nausea, loss of hearing, renal damage
Sulfonamides	Static	Broad (Gram+, Gram–)	Allergic responses, renal and hepatic injury, anemia
Tetracyclines	Static	Broad (Gram+, Gram–; Rickettsia and chlamydia)	Gastrointestinal upset, teeth discoloration, renal and hepatic injury
Trimethoprim	Cidal	Broad (Gram+, Gram–)	Allergic responses, rash, nausea, leukopenia
Vancomycin	Cidal	Narrow (Gram+)	Hypotension, neutropenia, kidney damage, allergic reactions

**Table 7:** Examples of cancer chemotherapy drugs Data reproduced with permission from [113], Springer Nature 2017.

Active Substance	Indication	Mechanism of Action	Safety Notes
Docetaxel	Breast cancer, non-small cell lung cancer	Increased assembly of microtubule	Mutagenicity positive; Carcinogenicity is not tested
Paclitaxel	Soft tissue tumor	Inhibition of microtubule reorganization	Mutagenicity positive; Carcinogenicity is not tested
Doxorubicin	Soft tissue tumor, ovarian tumor	DNA intercalation	Mutagenicity positive; Carcinogenicity is positive
Cyclophosphamide	Breast cancer; ovarian cancer	DNA intercalation	Mutagenicity positive; Carcinogenicity is positive
Docetaxel	Breast cancer, advanced stomach cancer	Microtubule network reorganization inhibition	Mutagenicity positive Carcinogenicity not tested
Epirubicin	Breast cancer	DNA intercalation	Mutagenicity positive; Carcinogenicity not tested
5-Fluorouracil	Head and neck cancer; breast cancer	Interferes with DNA replication	Mutagenicity positive; Carcinogenicity negative
Etoposide	Ewing’s sarcoma; uterine Cancer	Prevents re-ligation of the DNA strands	Mutagenicity positive; Carcinogenicity is limited
Rituximab	Follicular lymphomas	Bind to CD-20	Mutagenicity is not tested Carcinogenicity is not tested
Oxaliplatin	Colon cancer; rectal cancer	Interfere with DNA replication	Mutagenicity positive; Carcinogenicity positive
Ifosfamide	Ewing’s sarcoma, germ cell tumor	Interfere with DNA replication	Mutagenicity positive; Carcinogenicity positive

**Table 8:** Anticancer and antioxidant assay results on various plant extracts and herbs and spices. Data reproduced with permission from [138], Academic Journals 2012.

Scientific Name	Anticancer Activity		Antioxidant Activity	
	Water	Ethanoic	Ethanoic	Water
*Atriplex* sp.	100	49	70.8	50.5
*Euphorbia paralias* L.	3.3	2.4	81.1	51.8
*Cakile maritime* scop.	89.7	90.8	56.3	55.6
*Panax quinquefolius*	64	2.6	11.7	56
*Zygophulum album* L.F	61.1	32.9	80.3	64.8
*Asparagus stipularis*	13	5.2	72.7	70.9
*Kochia indica wight*	2.88	1.6	50.4	72.4
*Retama raetam* (Forssk) *Webb*	2.6	1.4	80.2	78.1
*Olea europaea* L.	0	8.0	50.5	81.1
*Pituranthos tortusous*	11.2	14.3	58.4	81.4
*Limoniastrum monopetalum* (L.) *Boiss*	52.9	3.8	85.6	82
*Cistanche phelypaea* (L.)	37	100	50.7	85.6
Moricandia nitens	89.2	51	89.8	85.6
*Zygophulum simplex* L.	61.1	32.9	85.7	44.1
*Arum palaestinum*	97.3	19.4	12.7	43.1
*Anabasis artiaulata* (Forssk.) *Moq*	25	10	40.8	42.7
*Thymelaea hirsute* (L.) *Endl.*	54	18	78.6	35.3
*Astragalus pinosus.*	100	15.8	28.4	19.5
*Asphodelus microcarpus salzm*	9.1	1.9	60.3	49.5
*Solanum nigrum*	100	89.7	85.7	55.6
*Lotas polyphylles*	7.2	7.9	27.0	27.0
*Beta vulgaris*	64	7.0	41.1	30.3
**Herbs and spices**				
*Rosmarinus oficinalis*	80.0	61	38.4	65.1
*Camellia sinensis*	85	86.4	85.4	70.6
*Cockatiel*	9.8	22.9	56.7	71.4
*Punica granatum*	6.1	4	85.7	75.8
*Glycyrrhiza glabra*	36	81	47.4	84.1
*Capsicum annuum*	24.4	68.6	57.3	25.0
*Ocimum basilicum*	77.2	76.3	72.3	9.8
*Zingiber officinale*	47.8	4.9	55.9	35.5
*Curcuma longa*	39.4	72.4	6.4	43.4
*Cassia italca*	89.7	90.78	55.4	30.7
*Nigella sativa*	81	2.54	8.4	8.8
*Solenostemma argel*	24.66	95	41.3	7
*Parviflora*	7.83	1.55	42.7	40.3

**Table 9:** Examples of nanoencapsulated phenolic compounds with anticancer, antibacterial and antioxidant properties. Some polysaccharide nano-encapsulating matrices were also surface modified either by polymeric coating or functionalization shown under “Wall structure” column. NPs signify nanoparticles. Data reproduced with permission from [143], Elsevier 2016 and [144] Elsevier 2021.

Matrix	Phenolic Compound	Wall Structure	Fabrication	Size Range (nm)	Target Application	Ref.
Cylocdextrin NPs	Curcumin & doxorubicin	Chitosan/poly(butyl cyanoacrylate)	Acidic anionic polymerization	130–135	Anticancer drug release	[146]
Cylocdextrin NPs	Catechin	Chitosan/poly(-glutamic acid)	Polyelectrolyte self-assembly	140–150	Controlled antioxidant release	[147]
Cylocdextrin NPs	Curcumin	Poly(butyl) cyanoacrylate (PBCA)/chitosan	Polymerization	200	Prevention of hepatic carcinoma with antiangiogenic effects	[148]
Cylocdextrin nanomicelles	Curcumin	β-lactoglobulin/alginate	Nano-suspension protein complexation	280	Sustained nutraceuticals delivery	[149]
Cylocdextrin NPs	Cathecin	β-cyclodextrin	Inclusion complex	67–470	Sustained antioxidant delivery	[150]
Cylocdextrin NPs	Oleoresin	Hydroxypropyl β-cyclodextrin	Inclusion complex	100–105	Sustained antibacterial delivery	[151]
Nano-starch	Quercetin	Cross-linked sodium trimetaphosphate	Self-assembly technique	20–40	Delivery through epithelium absorption	[152]
Micro-starch	Polyphenols from Hibiscus sabdariffa	Octenyl succinic anhydride	High shear homogenization	500–800	Sustained antibacterial effect	[153]
Micro-starch	Resveratrol	n/a	Solvent precipitation	500–800	Sustained antibacterial effect	[154]
Nano-starch	Curcumin	Polyvinyl alcohol	Sol-gel transformation	300	Controlled delivery of curcumin in cancer prevention	[155]

**Table 10:** Mathematical model parameters obtained from TCH release data as a function of drug loading concentration. Data reproduced with permission from [165], Elsevier 2022.

Amount of Drug (%)	0.5			1			3			5		
Models	Intercept	Slope	*R* ^2^	Intercept	Slope	*R* ^2^	Intercept	Slope	*R* ^2^	Intercept	Slope	*R* ^2^
Zero order	32.53	0.285	0.8642	34.9	0.332	0.8836	26.07	0.192	0.8298	17.77	0.138	0.8324
First order	3.47	0.006	0.7288	3.55	0.006	0.7679	3.25	0.005	0.7192	2.86	0.006	0.72
Higuchi	20.39	4.34	0.9724	20.95	5.023	0.9836	17.59	2.969	0.9636	11.68	2.131	0.9649
Korsmeyer-Peppas	1.28	0.262	0.9843	1.32	0.268	0.989	1.21	0.236	0.9906	1.03	0.245	0.9909

**Table 11:** Cancer drug release kinetic parameters for various nanofiber compositions. Data reproduced with permission from [166], Wiley 2018.

Nanofiber Formulations		pH	Korsmeyer-Peppas Parameters			Mechanism of Release
PCL:Chitosan	Percent Drug (5FU)		*n*	*a*	*R^2^*	
69:31	1	7.4	0.136	0.523	0.94	Fickian diffusion
		4.4	0.1595	0.501	0.93	
77:23	1	7.4	0.149	0.490	0.94	Fickian diffusion
87:13	1	7.4	0.143	0.481	0.93	Fickian diffusion
93:7	1	7.4	0.111	0.476	0.86	Fickian diffusion
100:0	1	7.4	0.370	0.151	0.96	Fickian diffusion

**Table 12:** Release kinetic parameters and mechanisms of sCT in different stimulated media. Data reproduced with permission from [167], Elsevier 2019.

Release Medium	Model	Equation	*R* ^2^	Release Kinetic	Mechanism
Simulated gastric fluid (SGF)	First order	*Ln(*1 *− Q) = −*0.01623 *t −* 0.08337	0.81752	−0.01623	Fickian diffusion
	Higuchi	*Q =* 0.05996 *t*^1*/2*^ *+* 0.02079	0.96895	0.05996	
	Weibull	*LnLn [*1/(1 *− Q)] =* 0.44996*Lnt −* 2.4400	0.93381	0.44996	
	Ritger-Peppas	*LogQ =* 0.42334*logt −* 1.07969	0.92736	0.42334	
Simulated intestinal fluid (SIF)	First order	*Ln(*1 *− Q) = −*0.03724 − 0.12604	0.83910	−0.03724	Fickian diffusion
	Higuchi	*Q =* 0.11943 *t*^1*/2*^ *+* 0.00116	0.97276	0.11943	
	Weibull	*LnLn[*1*/(*1 *− Q)] =* 0.59929*Lnt −* 2.0242	0.91437	0.59929	
	Ritger-Peppas	*LogQ =* 0.54001*logt −* 0.91097	0.89799	0.54001	
Simulated colonic fluid (SCF)	First order	*Ln(*1 *− Q) = −*0.12648 *–* 0.13138	0.96725	−0.12648	Case II transport
	Higuchi	*Q =* 0.19442 *t*^1*/2*^ *+* 0.08698	0.95482	0.19442	
	Weibull	*LnLn[*1*/(*1 *− Q)] =* 0.82806*Lnt −* 1.5129	0.98481	0.82806	
	Ritger-Peppas	*LogQ =* 0.5275*logt −* 0.67359	0.92158	0.5275	

**Table 13:** The coefficient of determination (*R*^2^) values of samples calculated by using different kinetic models. Data reproduced with permission from [168], American Chemical Society 2022.

Kinetic Model	3%Chit/30%CD- Cur-IC (pH 7.4)	3%Chit/30%CD- Cur-IC (pH 5.4)	2%Chit/2%Pect/ 30%CD-Cur-IC (pH 7.4)	2%Chit/2%Pect/ 30%CD-Cur-IC (pH 5.4)
Zero-order	0.4992	0.6901	0.5070	0.2005
First-order	0.7359	0.9485	0.7923	0.2181
Higuchi	0.7135	0.8461	0.7065	0.3727
KorsmeyerPeppas	0.6955	0.7148	0.6397	0.7271
Diffusion exponent (*n* value)	0.3813	0.4131	0.3473	0.3971
Hixson-Crowell	0.6602	0.9574	0.6912	0.2121

**Table 14:** Parameters obtained by fitting the quercetin release profiles to different release models. Note that *M_t_*/*M*_∞_ signifies the fraction released in time *t_i_*. Data reproduced with permission from [177], Elsevier 2018.

Model	Parameter	Pea Starch	Potato Starch	Corn Starch
Peppas-Sahlin $\frac{M_{t}}{M_{\infty }}=k_{1}t^{m}+k_{2}t^{2m}$	*R* ^2^	0.994	0.997	0.997
	AIC	37.980	29.910	22.878
	*k*_1_	20.868	10.740	19.305
	*k*_2_	−1.857	−0.493	−2.071
	*m*	0.325	0.457	0.226
Weibull $\frac{M_{t}}{M_{\infty }}=1-\exp\left[\frac{-{\left(t-T_{i}\right)}^{\beta }}{\alpha }\right]$ *T_i_*: Lag time. *β*: A constant related to the shape of the dissolution curve *α*: Scale parameter that defines the time scale.	*R* ^2^	0.994	0.984	0.997
	AIC	37.402	49.867	21.800
	*α*	2.845	5.033	4.379
	*β*	0.178	0.289	0.160
	*T_i_*	4.342	3.493	2.651
Korsmeyer-Peppas $\frac{M_{t}}{M_{\infty }}=Kt^{n}$ *K*: A constant that depends on the dosage form characteristics. *n*: Release exponent that indicates the release mechanism	*R* ^2^	0.976	0.966	0.995
	AIC	51.299	56.493	25.911
	*k*	25.791	17.067	19.261
	*n*	0.161	0.240	0.146
Higuchi $\frac{M_{t}}{M_{\infty }}=kt^{1/2}$ *k*: Higuchi dissolution constant.	*R* ^2^	0.211	0.629	0.117
	AIC	87.719	80.627	80.265
	*k*	5.258	4.993	3.660
Baker-Lonsdale $\frac{3}{2}\left[1-{\left(1-\frac{M_{t}}{M_{\infty }}\right)}^{\frac{2}{3}}\right]-\frac{M_{t}}{M_{\infty }}=k_{BL}t$ *k_BL_*: A release constant.	*R* ^2^	0.480	0.783	0.289
	AIC	83.135	74.736	77.889

**Table 15:** The fitted equations and the coefficient of determination (*R*^2^) of CAP release kinetic models in free form and from prepared CAP-IRSNPs in 50% ethanol solution, PBS of 1.2 and 7.0. CAP: capsaicin; CAP-IRSNPs: capsaicin-loaded indica rice starch nanoparticles. Data reproduced with permission from [181], Elsevier 2022.

Release Medium	Empty Cell	Mathematical Model	CAP	CAP-IRSNPs
50% ethanol solution	Zero-order kinetics model	*R* ^2^	0.425	0.551
	First-order kinetics model	*R* ^2^	0.999	0.992
	Higuchi model	*R* ^2^	0.748	0.807
	Korsmeyer-Peppas model	*R* ^2^	0.995	0.873
		*n*	0.032	0.366
	Hixson-Crowell equation	*R* ^2^	0.744	0.830
PBS of 1.2	Zero-order kinetics model	*R* ^2^	0.155	0.688
	First-order kinetics model	*R* ^2^	0.996	0.996
	Higuchi model	*R* ^2^	0.293	0.878
	Korsmeyer-Peppas model	*R* ^2^	0.557	0.899
		*n*	0.111	0.434
	Hixson-Crowell equation	*R* ^2^	0.379	0.961
PBS of 7.0	Zero-order kinetics model	*R* ^2^	0.524	0.461
	First-order kinetics model	*R* ^2^	0.996	0.997
	Higuchi model	*R* ^2^	0.675	0.836
	Korsmeyer-Peppas model	*R* ^2^	0.828	0.887
		*n*	0.228	0.347
	Hixson-Crowell equation	*R* ^2^	0.689	0.863

**Table 16:** Applied mathematical models to the release data of pectin nanoparticles (upper line) and NIMs (polymer shell coated nanoparticles-lower line) with their statistical evaluation. Data reproduced with permission from [182], Elsevier 2021.

Model *	Equation	*R* ^2^	*R* ^2^_adjusted_	AIC	MSC	n
First-order	$F=100\left(1-e^{-k1t}\right)$	0.7974 0.8280	0.7974 0.8280	66.63 39.04	0.8394 0.9753	– –
Hixson-Crowell	$F=100\left[1-{\left(1-k_{\mathrm{HC}}t\right)}^{3}\right]$	0.6895 0.7167	0.6895 0.7167	70.48 42.03	0.4124 0.4763	– –
Higuchi	$F=k_{H}t^{0.5}$	0.8749 0.9730	0.8749 0.9730	62.30 27.92	1.321 2.8287	– –
Hopfenberg	$F=100\left[1-{\left(1-k_{\mathrm{HB}}+5\right)}^{n}\right]$	0.7140 0.7619	0.6731 0.7024	71.74 42.99	0.2722 0.3167	– –
Korsmeyer-Peppas	$F=k_{\mathrm{KP}}t^{n}$	0.9969 0.9937	0.9961 0.9921	16.87 21.19	4.378 3.9503	0.376 0.430

* In all models: *F* is the fraction (%) of drug released in time t. *k*_1_: first-order release constant. *k*_HC_: Hixson-Crowell release constant *k*_H_: Higuchi release constant. *k*_HB_: Hopfenberg release constant. *k*_KP_: release constant incorporating structural and geometric characteristics of the drug-dosage form. *n* is the diffusional exponent indicating the drug-release mechanism.

**Table 17:** Mathematical models and parameters based on release data. The data is reproduced with permission from [185], Hindawi Publishing.

	Zero-Order	First-Order	Higuchi’s	Hixson-Crowell		Korsmeyer-Peppas		Kopcha
pH	*R* ^2^	*R* ^2^	*R* ^2^	*R* ^2^	*R* ^2^	*n*	*R* ^2^	*A/B*
3.0	0.77	0.81	0.93	0.80	0.80	0.28	0.89	36.87
4.0	0.76	0.82	0.93	0.80	0.80	0.30	0.93	37.12
5.0	0.81	0.89	0.96	0.87	0.87	0.34	0.97	43.08
6.0	0.76	0.82	0.93	0.80	0.80	0.30	0.92	36.93
7.4	0.75	0.79	0.92	0.78	0.78	0.31	0.93	36.91

**Table 18:** Classification of polysaccharide-based nanoparticles as carriers for drug delivery and release. Data compiled from [223] no written permission required TUOMS PRESS, Tabriz University of Medical Sciences.

Nanoparticle System	Physicochemical Properties	Comments	References
Chitosan	Shows mucoadhesive properties and ability to open tight junctions between epithelial cells	Its cationic nature mediatesdelivery of negative molecules such as DNA	[224,225]
Alginate	Shows mucoadhesive and gelling properties	Its anionic nature mediates deliveryof cationic agents	[226,227]
Heparin	An anionic and highly sulfated polysaccharide that shows anticoagulant properties	Ideal system for delivery of growthfactor	[228,229]
Hyaluronic acid	Affinity to water absorption and gel forming	Facilitates passive tumor targeting through CD44 receptor-mediated endocytosis	[230,231]
Dextran	A neutral polysaccharide with lower cytotoxicity	Degradation of nanoparticles occursby dextranase	[232]
Pulluan	A neutral polysaccharide produced by a specific fungus	Relative high cost of pullulan haslimited its application	[233]
Pectin	An anionic polysaccharide with gelling and film forming ability	Nanoparticles are degraded by pectinase secreted by bacteria present in the large intestine	[234,235]

## Data Availability

Not applicable.
